# A Multiscale Approach to Modelling Drug Metabolism by Membrane-Bound Cytochrome P450 Enzymes

**DOI:** 10.1371/journal.pcbi.1003714

**Published:** 2014-07-17

**Authors:** Richard Lonsdale, Sarah L. Rouse, Mark S. P. Sansom, Adrian J. Mulholland

**Affiliations:** 1 Centre for Computational Chemistry, School of Chemistry, University of Bristol, Bristol, United Kingdom; 2 Department of Biochemistry, University of Oxford, Oxford, United Kingdom; Wellcome Trust Sanger Institute and European Bioinformatics Institute, United Kingdom

## Abstract

Cytochrome P450 enzymes are found in all life forms. P450s play an important role in drug metabolism, and have potential uses as biocatalysts. Human P450s are membrane-bound proteins. However, the interactions between P450s and their membrane environment are not well-understood. To date, all P450 crystal structures have been obtained from engineered proteins, from which the transmembrane helix was absent. A significant number of computational studies have been performed on P450s, but the majority of these have been performed on the solubilised forms of P450s. Here we present a multiscale approach for modelling P450s, spanning from coarse-grained and atomistic molecular dynamics simulations to reaction modelling using hybrid quantum mechanics/molecular mechanics (QM/MM) methods. To our knowledge, this is the first application of such an integrated multiscale approach to modelling of a membrane-bound enzyme. We have applied this protocol to a key human P450 involved in drug metabolism: CYP3A4. A biologically realistic model of CYP3A4, complete with its transmembrane helix and a membrane, has been constructed and characterised. The dynamics of this complex have been studied, and the oxidation of the anticoagulant R-warfarin has been modelled in the active site. Calculations have also been performed on the soluble form of the enzyme in aqueous solution. Important differences are observed between the membrane and solution systems, most notably for the gating residues and channels that control access to the active site. The protocol that we describe here is applicable to other membrane-bound enzymes.

## Author Summary

A significant amount of information about how enzymes and other proteins function has been obtained from computer simulations. Often, the size of the system that is required to provide a sufficiently realistic model places limitations on both the timescale of the simulation, and the level of detail that can be studied. Computational approaches that utilise more than one type of method (so-called ‘multiscale methods’) allow the size of system, and timescale of study, to be increased. Membrane-bound proteins, such as cytochrome P450 enzymes (P450s), are an example of where multiscale simulations can be used. P450s are important in drug metabolism, and are known to be involved in adverse drug reactions. Access of substrate to the active site of these enzymes through the membrane is not well-understood. In the present work, a simulation pipeline is presented that leads through the construction and refinement of a realistic protein:membrane system by molecular dynamics simulations to reaction modelling. An important drug-metabolising P450, CYP3A4, is used as an example, together with the anticoagulant drug R-warfarin. Our investigations reveal that a membrane-bound model is required to fully capture the gating mechanisms and substrate ingress/egress channels. The simulation protocol described here is transferrable to other membrane-bound proteins.

**This is a *PLOS Computational Biology* Methods Article**

## Introduction

Molecular simulation methods are widely used to study membrane proteins. [1] An advantage of these methods is that the protein of interest can be studied in an approximately native environment. A limitation is imposed by the spatial and temporal scales accessible at a single level of description. In many cases, it may be useful or necessary to investigate levels of detail ranging from protein-lipid interactions on long timescales, to chemical reactions and electronic structure. This type of application is particularly well exemplified by the cytochrome P450 enzymes, where the questions of adverse drug interactions and of substrate access are still not well-understood. However, multiscale modelling is extending the scope of computational methods to overcome this limitation and further the understanding of biological processes. For example, coarse-grained molecular dynamics simulations, in combination with atomistic simulations (CG/AT), allow the study of larger systems for longer timescales than those accessible by atomistic simulations alone. [2] Similarly, hybrid quantum mechanics/molecular mechanics (QM/MM) methods enable the calculation of reaction mechanisms in enzymes to high accuracy, whilst explicitly including the effects of the surrounding enzyme and solvent environment. [3] In order to model the reactions of enzymes in large biological assemblies, multiscale methods, which span the range from CG through AT up to the QM/MM level, will allow us to answer these important questions in chemical biology. The framework that we introduce here allows these questions to be answered.

Simulations of membrane proteins provide an example application of where multiscale techniques can help us to understand the relationship between structure and function in biological molecules. Membrane proteins are involved in many biological process, such as transport, signalling, and enzymatic activity. The cytochrome P450 enzymes (P450s) are members of the latter category and perform a variety of functions, such as steroid synthesis and drug metabolism. [4] Whilst P450s in prokaryotes are soluble proteins (such as CYP101 and CYP102A1), eukaryotic P450s are membrane-bound. Binding to the membrane occurs via an N-terminal transmembrane *α* helix, and enables the protein to be oriented close to its redox partner protein, which in the case of human P450 isoforms is cytochrome P450 reductase. [5], [6] The redox partner protein is required in order to supply the two electrons that are necessary for the reaction cycle. Significant challenges are posed by the modelling of drug metabolism by P450s, particularly in predicting metabolite formation. Simulation methods, such as docking, molecular dynamics, and QM/MM modelling have been shown to be useful in rationalising the selectivity of oxidation of substrates. [7]–[9] However, other important factors, such as substrate access (via the membrane) and binding to the active site, must be considered for a more complete perspective on how drugs are metabolised.

To date, all of the human P450 structures that have been determined by X-ray crystallography are for truncated enzymes, from which the transmembrane helix has been deleted, in order to increase the solubility of the protein. [10]–[12] However, typical P450 substrates involved in drug metabolism are hydrophobic. Indeed, it is the oxidation of such substrates by P450s that increases the solubility of hydrophobic drug molecules to aid excretion. It has been suggested that the entrance of the substrate into the active site of the drug metabolising P450s occurs via the membrane. [13] Hence, it follows that to study the entrance of substrates into human P450s, the membrane environment should be included. Unless P450s can be crystallized in their membrane-bound form, modelling approaches are thus required. In the present study we focus on drug metabolism, but similar approaches will also be applicable to e.g. biocatalysis and signalling.

Atomistic MD simulations of membrane-bound CYP3A4 have been performed previously using a lipid bilayer consisting of 1-palmitoyl-2-oleoyl-sn-glycero-3-phosphocholine (POPC). [14] The protein was placed into the bilayer at a depth consistent with the Orientations of Proteins in Membranes (OPM) database. [15]. A stable pathway was found, linking the active site and membrane through the region between the B-C loop and the beta domain. The presence of the membrane was found to affect the opening and closing of the tunnels that link the active site to the surrounding environment. Membrane-bound CYP3A4 has also been simulated using a simplified model of a lipid bilayer to enhance lipid mobility. [16] The authors observed spontaneous binding and insertion of the globular domain of the enzyme into the membrane mimetic during 50 ns unbiased simulations. The transmembrane helix was not present in the majority of the membrane-bound simulations. Membrane binding was found to induce conformational changes on the protein at the membrane interface, causing changes to the active site access channels, compared to the crystal structure and a single simulation performed in solution. In the same study, experimental measurements of the heme tilt angle were performed for CYP3A4 bound to a nanodisc membrane. The average tilt angle was measured as 60°, which is in good agreement with the previously measured angles for CYP17A1 (47–63°) and CYP21A2 (38–78°). [17] The tilt angle calculated from the simulations of CYP3A4 was in the range 70–80°.

Coarse-grained molecular dynamics (CGMD) simulations are widely exploited for building models of protein:membrane systems. [18] CGMD allows the orientation of a protein within a membrane to be determined, whilst taking into account specific interactions between the lipid headgroups and hydrophobic tails. Conversely, these interactions are not taken into account in methods where the membrane is treated as a hydrophobic ‘slab’ (such as OPM). CGMD simulations can be used to generate configurations for conversion to atomistic models, for subsequent study using atomistic molecular dynamics simulations. [2] This type of methodology has been applied previously to CYP2C9. [19] A model of the transmembrane helix was constructed and incorporated into a lipid bilayer via a CGMD self assembly simulation. The protein was subsequently attached to the transmembrane helix and further CGMD simulations were performed. It was necessary to perform these two steps separately, because in test simulations of the self-assembly of the bilayer around the complete protein and transmembrane helix, the bilayer did not assemble around the transmembrane helix. The resultant CG protein:membrane model was used as the basis of atomistic molecular dynamics simulations. The presence of the membrane was found to have a limited effect on the conformational flexibility of the protein, and was localised to the regions that made contact with the lipid bilayer. The authors found more than one possible orientation of the CYP2C9 in the membrane, differing by the conformation of the FG loop. The membrane-bound CYP2C9 remained in closed or almost-closed conformations throughout the simulations, however, motions were observed that corresponded to the opening and closing of tunnels from the enzyme active site.

Whilst previous studies have shown that the membrane has an effect on the dynamics and active site channels of P450s, it is currently not known whether binding has an effect on the electronic structure of the key intermediates in the catalytic cycle, specifically the iron-oxo complex, Compound I (Cpd I). [20] Cpd I is widely accepted to be the active oxidizing species in P450s. Any changes in Cpd I may result in differences in reactivity (and selectivity) between membrane-bound P450s and those in solution.

In order to study electronic structure and chemical reactivity, a computational method is required that treats electrons explicitly, such as quantum mechanics (QM). In large systems, such as enzymes, it is convenient to partition a model system into QM and molecular mechanical (MM) subsystems, in order to make the calculation computationally feasible. The QM region comprises a region of sufficient size to model the chemical reaction of interest, typically of around 50–100 atoms. QM/MM calculations have been used to study many enzymatic reactions, including those in P450s. [8], [21]–[24] Such calculations have aided in the characterisation of intermediates in the catalytic cycle, and have provided mechanistic information concerning various oxidative reactions. The choice of QM method is an important one, and often a compromise between speed and accuracy is necessary. Density functional theory methods (in particular the B3LYP functional) have been identified as an effective compromise between speed and accuracy for calculations on Cpd I. [21], [24]–[28] In previous work, it has been shown that including an empirical dispersion correction in DFT calculations improves the accuracy of calculated barriers to oxidation, relative to those where the correction is not included. [29], [30]

Numerous studies of P450 mechanisms have been performed using gas phase QM models and QM/MM calculations. [24], [28], [31] Whilst gas phase calculations provide useful insight into the reactivity of P450s, inclusion of the local protein environment (e.g. using a QM/MM approach) provides a more realistic model and improves agreement with experiment. [8], [21] For example, the electronic structure of Cpd I has been found to be sensitive to its local environment: the distribution of unpaired electron spin density differs between vacuum and QM/MM models. [25] Similarly, when calculating activation energy barriers for hydroxylation and epoxidation in P450s, better agreement with experiment is observed when using QM/MM, compared to QM calculations performed on a gas phase model system. [21] It is not currently known whether further extension of the size of MM part of the QM/MM model to include the transmembrane helix and membrane, would further improve the accuracy of P450 calculations. The heme in P450s is relatively far away from the membrane and hence any electrostatic effects of the membrane on the heme are unlikely to be significant. However, MD simulations of membrane-bound P450s have suggested that the dynamics of P450s are influenced by the presence/absence of membrane. [16], [32] Therefore, it is conceivable that subtle changes of the protein environment surrounding Cpd I may occur due to the presence of the membrane.

**Figure 1:**
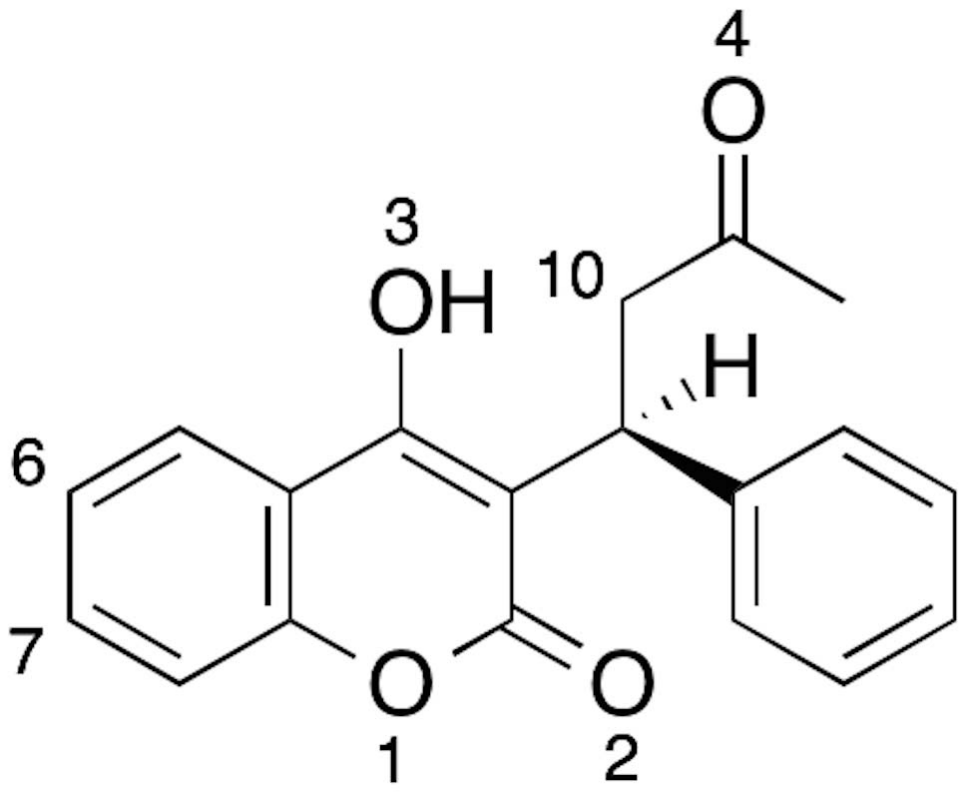
Chemical structure of R-warfarin. In CYP3A4, R-warfarin undergoes hydroxylation at C10. [34]

This study is the first application of a simulation pipeline that spans the range from CGMD, to atomistic MD simulations, to QM/MM reaction modelling. Other integrated workflows are currently being developed for making better predictions of P450-related toxicity. [33] In the current work, we have studied CYP3A4, as it is responsible for the metabolism of the majority of therapeutic drugs, and is relatively well-studied in terms of its membrane localization and orientation, as described above. [14], [16] We have chosen to model the substrate R-warfarin (Fig. 1), a widely used anticoagulant drug with a narrow therapeutic index due to drug-drug interactions, and a known substrate of CYP3A4. [34] R-warfarin undergoes aliphatic hydroxylation at C10. [34] The hydroxylation of S-warfarin in CYP2C9 has been studied previously by QM/MM using a truncated enzyme model in the absence of membrane and transmembrane helix. [8] In that work it was shown that QM/MM calculations are capable of rationalising the regioselectivity of oxidation by P450s, and were better at doing so than performing calculations using gas phase models consisting solely of Cpd I and substrate.

We find that the presence of the membrane significantly affects the gating residues that control access to the active site of the protein. However, the presence of the membrane does not seem to influence residues surrounding the active oxidising species, and hence the reactivity of this species is unchanged. This finding provides an important validation for the use of engineered P450s as models of their *in vivo* counterparts.

## Results

### Coarse-grained molecular dynamics simulations

Coarse-grained MD simulations were used in two stages to generate a stable model of the membrane-bound form of the protein in a simple model of the endoplasmic reticulum (POPC/POPE) lipid bilayer. The first set of simulations was used to determine the relative orientations of the transmembrane (TM) and globular/soluble domains. This is a necessary requirement of using CGMD simulations where the secondary structure is predefined (usually based on the original atomistic structure), and tertiary structure is generally maintained by using an elastic network. CGMD simulations were performed, in which the restraints between the TM and globular domains were relaxed (see Fig. S1 and Methods), allowing the protein to equilibrate while the membrane self-assembled. In each case, the TM domain inserted into the membrane. The most representative model, based on structural clustering of this first set of trajectories, was then used as the starting point for further CGMD simulations, in which the globular/TM orientation and secondary structure was restrained.

**Figure 2:**
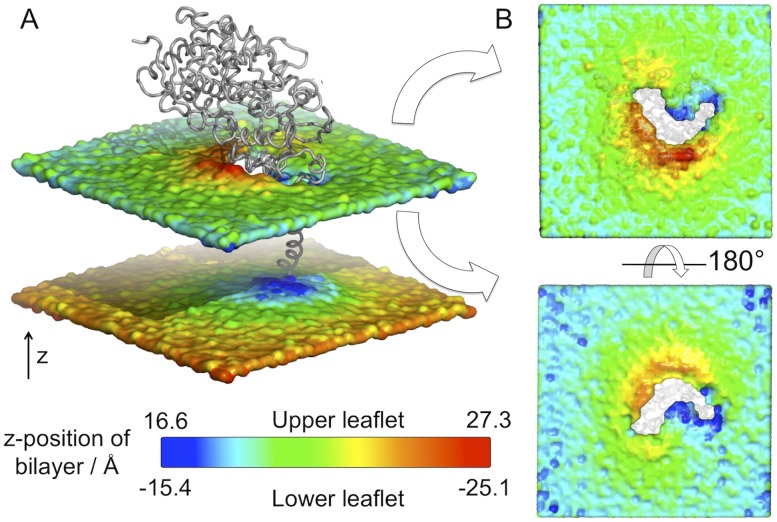
Membrane deformation about CYP3A4. The positions of the phosphate particles of all lipids relative to the position of the protein (shown as a grey backbone trace) averaged over a 1 *μ*s coarse-grained molecular dynamics simulation are displayed as a surface. The surface is coloured according to the position along the normal to the bilayer plane (Z-position) with red corresponding to large distances from the bilayer centre and blue corresponding to the smallest distances from the bilayer centre. Thus, red areas correspond to regions of the bilayer that thicken to accommodate the presence of the protein, whilst blue regions correspond to regions of bilayer thinning. A: Side view of the protein within the bilayer. B: Views from above and below the upper leaflet, highlighting the asymmetric deformation of the bilayer. The membrane is observed to deform asymmetrically, with the membrane thinning (blue) in the region of the A-anchor and thickening (red) in the region of the F′- and G′-helices. The lower leaflet is not shown and the protein is transparent for clarity.

In each of the second set of CGMD simulations, the TM domain adopted a transmembrane orientation. In agreement with simulations of CYP3A4 with a HMMM membrane model, [16] the A-anchor (residues 44–47) was found to insert most deeply into the hydrophobic region of the membrane, followed closely by the G′-helix. The F′-helix formed hydrophobic contacts with the lipid tail groups, but to a lesser extent than observed previously [16] (Fig. S2). The bilayer was observed to thin to accommodate the A-anchor and G′ region, and thicken on the opposite side near the F′ and FG loop (Fig. 2). Specific lipid binding sites were observed in the A anchor region. Preliminary simulations of the globular domain alone interacting with the membrane led to symmetrical thickening of the bilayer (unpublished data), implying that the presence of the trans-membrane helix affects deformation of the bilayer and, therefore potentially, substrate access.

A comparison of the averaged lipid headgroup positions from these simulations with the predicted membrane-bound orientation from the OPM database is revealing (Fig. S3). There is a ∼20° difference in the orientation of the membrane normal relative to the protein in the two cases. However, close to the interaction surface of the protein, the membrane is distorted in the CGMD simulations, such that the interacting regions agree well between simulation and the OPM model based on experimental data.

The orientation of the globular domain may be fully defined in terms of its depth of insertion in the membrane, as well as by choosing two orthogonal vectors 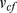 and 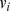 and measuring the angle between the membrane normal and each of these (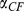 and 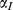 respectively). [16]. The vectors 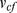 and 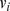 are defined as follows: 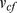 connects one helical turn in each of helix C and helix F (the midpoints of the 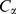 atoms of residues 137–141 and 207–211, respectively). v*_i_* is orthogonal to 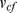, and connects the centres of the first and last helical turns in helix I (midpoints of *C*_α_ atoms of residues 292–296 and 321–325, respectively). These properties are observed to equilibrate within the first 30 ns and converge over the 4 simulations (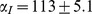
°, 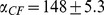
°). The unbiased CGMD simulations therefore lead to a single, conserved orientation in the the membrane.

The configuration generated from the CGMD simulations was converted to an atomistic model (see Methods) for further simulations, described in the sections that follow.

### Atomistic molecular dynamics simulations

Four different model systems were simulated with atomistic MD: the membrane-bound and soluble forms of CYP3A4, both with and without R-warfarin present in the active site (details of the setup for all models is provided in the Methods section). Three 50 ns simulations were performed for each system. Unlike the coarse-grained methods, atomistic simulation allows tertiary structure change and so a degree of conformational relaxation was expected, and is indeed important in allowing conformational changes associated with membrane binding. Some initial relaxation occurs, with the orientation of the protein converged by the final 20 ns of simulations. Hence only the last 20 ns of each simulation was used for analysis. The root mean square deviation (RMSD) of the backbone alpha carbon atoms from their average positions gives an overall indication of the stability of each system during a simulation. The RMSD is very similar between the membrane and solution simulations, with values ca. 1.6 Å and ca. 2.1 Å, respectively. The orientation of the protein in the bilayer generated using this unbiased simulation approach may be compared to calculated orientations of the heme group of CYP3A4 from linear dichroism experiments [16] as discussed in the Introduction. The average heme tilt angle calculated from atomistic simulations is 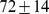
°. This compares to the experimental value (calculated for CYP3A4 bound to a nanodisc) of 60 and previous simulation value of 72°. [16] There are no notable losses of secondary structure in any of the membrane or solution simulations (see Fig. S4). The multiscale CG/AT approach therefore provides a well-equilibrated starting point for further simulation.

#### Membrane-protein interactions

**Figure 3:**
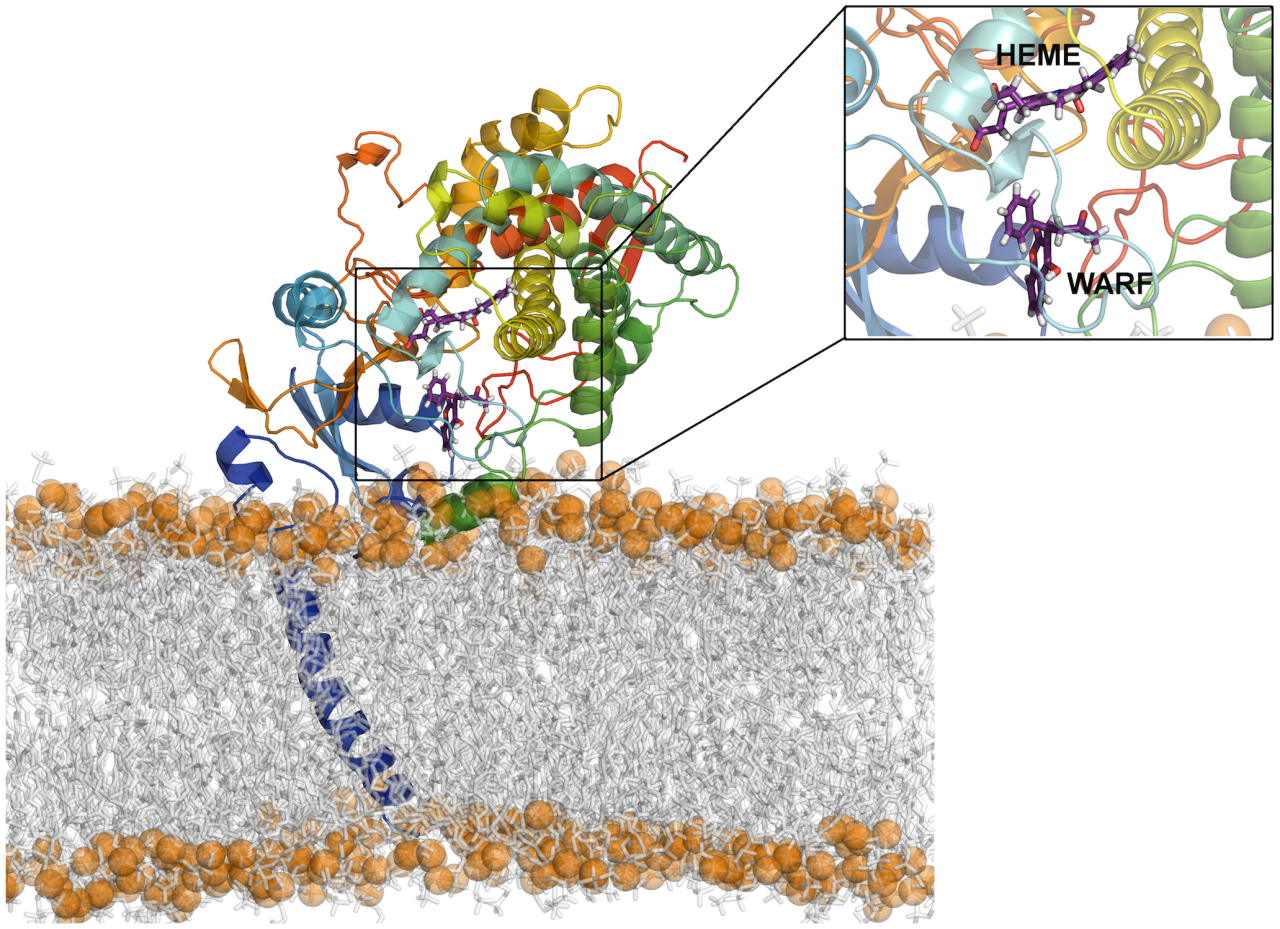
Positioning of CYP3A4 in the lipid bilayer from atomistic MD simulations. This configuration was generated from unbiased coarse grained simulations, in which a mixed POPC/POPE bilayer was self-assembled around the protein. The transmembrane helix spans the bilayer, with the A-anchor and F′ and G′ helices buried in the hydrophobic region of the membrane. This snapshot corresponds to the position of the protein with R-warfarin bound following conversion to full atomistic resolution, equilibration and simulation. Inset: active site region containing Compound I (Cpd I) and R-warfarin (WARF). The protein is shown in cartoon representation coloured by helix, lipids are shown in transparent grey stick representation, with the phosphate particles shown as orange transparent spheres. R-warfarin and Compound I are both shown in stick representation.

As discussed above, in addition to the transmembrane helix, several regions of the protein were buried in the membrane during the simulations. These regions (the A-anchor, and the F′ and G′ helices) were observed to remain buried during atomistic simulation (as shown in Fig. 3). Additionally, the highly flexible Gln200 next to the FG loop region (residues 202-260) region was found to interact with the membrane at various points in some simulations. This is in good agreement with simulations performed on membrane-bound CYP2C9. [19]

Conformational clustering analysis was performed to determine if the presence of the membrane led to any conformational changes (see Method section). C*_α_* RMSD comparison matrices of top representative structures from each simulation were calculated (Fig. S5). These showed that the soluble form of the protein was least affected by the presence or absence of bound warfarin. The membrane bound forms deviated most, however the maximum RMSD between all representative models was below 3.6 Å. Conformational clustering of the 141 residues making up the active site region led to similar patterns. However, the active site region typically only varied by ∼1 Å between the four sets of simulations, with a maximum deviation of 2.3 Å.

#### Effect of membrane on protein flexibility

The effect of the membrane on protein flexibility was assessed by comparing the root mean square fluctuations (RMSFs) of the backbone atoms over the course of all simulations, calculated relative to the average coordinates for the respective simulation (see Fig. S6). Generally, the RMSF profiles were similar between the membrane-bound and solution simulations. For simulations of CYP2C9 [19], the presence of the membrane was found also to have limited influence on the flexibility of the protein. The most notable difference between the two sets of simulations studied here (membrane-bound and solution) is observed between residues 180–215, corresponding to the F helix, and the EF and FF′ loops. Interestingly, this is a region of high B-factors in the crystal structure (see Fig. S6).

In the membrane-bound simulations, the transmembrane helix has a relatively high RMSF (∼4 Å), particularly for residues close to the N-terminus. No significant differences in RMSF are observed between the substrate-bound and apo models, for either the membrane-bound or solution simulations. We do not observe any major conformational changes when comparing the enzyme bound to the membrane with the enzyme in solution. The apo solution simulations have slightly increased RMS fluctuations in the region 90-110 that are not observed during the simulations of the other three models.

#### Substrate ingress/egress channels

**Figure 4:**
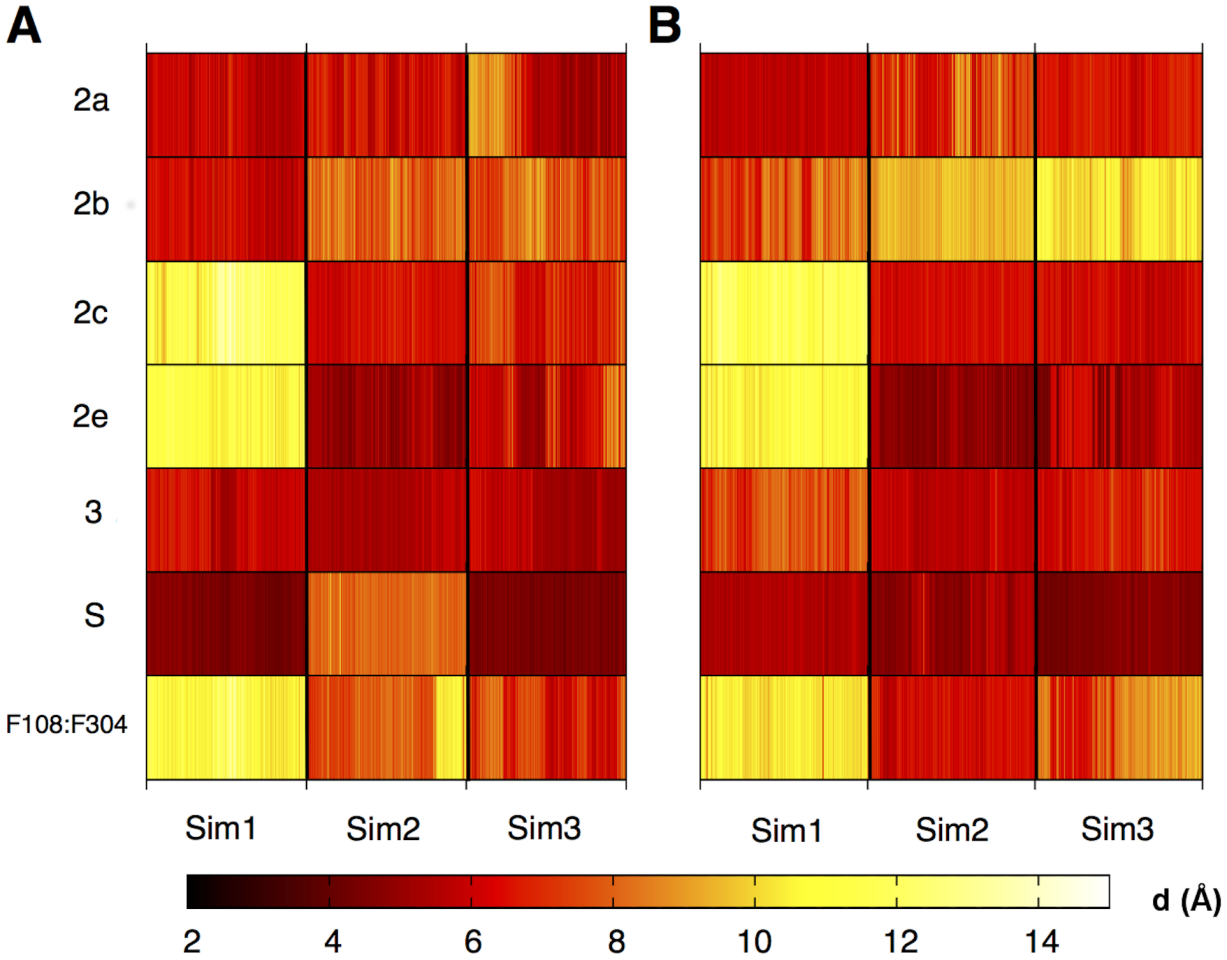
Opening and closing of substrate ingress channels over time for membrane simulations of CYP3A4. Distance between gating residues (d) [in Å] for simulations of membrane-bound CYP3A4 in the absence (A) and presence (B) of R-warfarin. The channels and gating residues are as described in Table 1. Distances were calculated at 0.02 ns intervals over the final 20 ns of each simulation. Similar patterns of opening/closing of gates are observed between the apo model (left) and the R-warfarin bound model (right).

**Figure 5:**
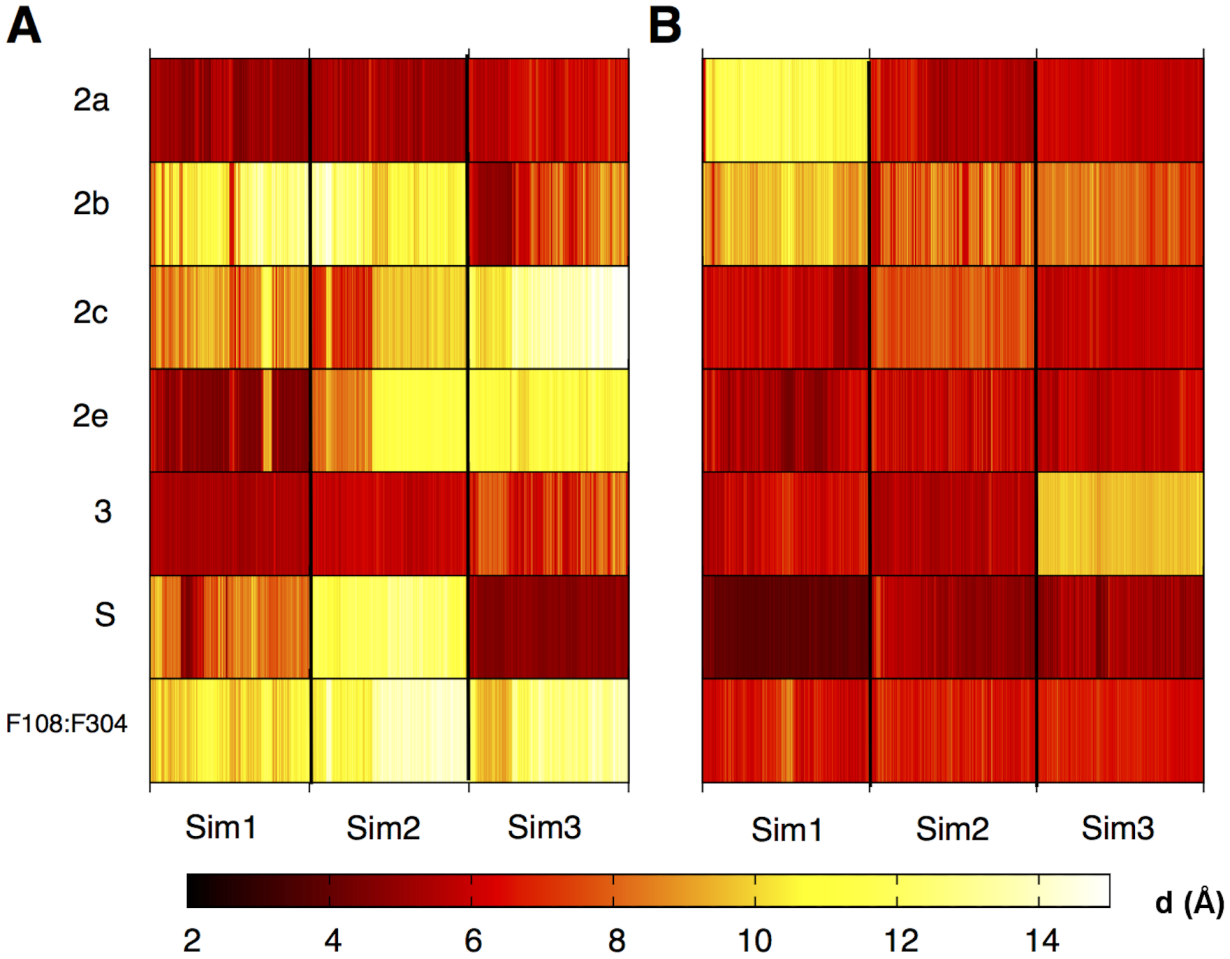
Opening and closing of substrate ingress channels over time for soluble simulations of CYP3A4. Distance between gating residues (d) [in Å] for simulations of soluble CYP3A4 in the absence (A) and presence (B) of R-warfarin. The channels and gating residues are as described in Table 1. Distances were calculated at 0.02 ns intervals over the final 20 ns of each simulation. The gating residues are generally more open in the apo simulations, compared to those where R-warfarin is bound. This is in contrast to the membrane-bound simulations (see 4), where the gating residues display similar behaviour for the apo and R-warfarin bound models.

Several channels have been identified in previous work [32] that are believed to allow the entrance and exit of substrate, product and solvent molecules. These channels have been identified as involved in the entrance and exit of substrates, products and solvent. [35] Each channel has an associated ‘gate’ formed by two side chains, which are generally phenylalanines. [36] These gating residues have been studied previously for the soluble form of CYP3A4 using MD simulations. [37] The nomenclature of these gates is provided in Table 1, along with the identities of the gating residues. The distances between these gates was monitored during the simulations. No single gate remained open or closed when considered across all simulations, supporting the hypothesis that they are indeed dynamic gating residues. The distances between gating residues for the membrane and water simulations are provided in Figs. 4 and 5, respectively (numerical values and errors are provided in Table S4). When comparing the number of open gates between the substrate complex and apo enzyme simulations (See Table S4), this is approximately equal (∼40%) for the membrane simulations. In the case of the solution simulations, there are significantly more open gates observed for the apo (∼60%) enzyme compared to the simulations of the enzyme containing R-warfarin (∼30%). The presence of the membrane therefore appears to modulate the opening and closing of gates.

**Table 1:** Substrate access channels in CYP3A4 and their corresponding gating residues.

Channel name	Gating Residues
2a	F57-F215
2b	F108-F220
2c	F108-F241
2e	F108-I120
3	F213-F241
S	R212-L482

Channel nomenclature assigned according to ref [32], where appropriate.

Preliminary analysis of the active site volumes found no significant difference between the apo and substrate-bound forms of the membrane-bound forms. The active site volumes were found to be variable between repeat simulations and within a given simulation. Consistent with the outcome of conformational clustering of the active site region, no clear trends are observed between the soluble/membrane bound or apo/subtrate bound forms. (Fig. S7) However, the soluble apo simulation had a significant increase in active site volume, presumably due to the increased channel opening.

In a previous study [16], a rearrangement of a cluster of Phe residues was observed in the membrane-bound form and was attributed to interactions with the membrane. This rearrangement was observed in both soluble and membrane-bound simulations described here, suggesting that the rearrangement is not membrane-induced. Channel 2e was found to close in membrane-bound CYP3A4 but was able to open in the soluble form, consistent with behaviour observed for CYP2C9 [14]. Fishelovitch et al. [35] have observed that binding of CYP3A4 to the reductase protein (cytochrome P450 reductase - CPR) is involved in the activation of water channels. Hence, it is possible that inclusion of CPR in the membrane-bound model may induce further opening/closing of channels, however, this is beyond the scope of the current work.

The substrate entrance/exit channels were investigated using CAVER 3.0 [38]. The origin used for tunnel calculations was the ferryl oxygen atom of Cpd I. Tunnels were calculated for each simulation at 5 ns intervals (Figs. S8–S9). An alignment of the Caver channels with the lipid bilayer distortion plots from the CGMD simulation allows us to identify which of these channels are in contact with the lipid headgroups (Fig. S10). In particular, the 3 tunnel between the F′–G′ helices points directly into the membrane. This is consistent with the hypothesis that the substrate may enter the enzyme from the membrane.

#### Conformation of Arg212

Arg212 has been identified previously as being important in the binding of substrates to CYP3A4. [39] Two different conformations of this residue are observed in the deposited crystal structures: the sidechain can point both into and out of the active site. [12], [40] In the crystal structure that was selected as the starting point for this work (PDB entry 1TQN), the side chain of Arg212 is directed into the active site. During the atomistic MD simulations, movement of this sidechain between the two conformations is observed, both for the membrane-bound and soluble systems and regardless of whether R-warfarin is present. As is described below, Arg212 is observed to interact with the substrate during the atomistic MD simulations, as described in the following section.

#### Mobility of warfarin in the active site

The RMSD of R-warfarin was calculated during the last 20 ns of each simulation of CYP3A4 containing warfarin (see Fig. S11). Differences are observed in the amount of movement of warfarin around the active site between simulations, due to the presence and absence of hydrogen bonds between warfarin and the active site residues, as described below.

In the three R-warfarin membrane simulations, different binding orientations of warfarin were observed, despite all three simulations starting from the same initial binding orientation. In one of these simulations (simulation 1 in Fig. S11), the substrate was located relatively close to Cpd I, with the two hydrogen atoms connected to C10 remaining within 6 Å of the ferryl oxygen. In this simulation, there is a hydrogen bond between the Arg212 sidechain and the carbonyl oxygen on the bicyclic scaffold of warfarin (O2 in Fig. 1). During this simulation, the gate formed by Phe108 and Phe304 is open, as shown in Fig. S12A. In simulation 2, warfarin is located further from Cpd I than in the simulation 1 (the center of mass of warfarin is an average of 2.4 Å further away from the heme than in the first simulation) and an Arg212 NH_2_ group forms a hydrogen bond with the aliphatic carbonyl oxygen of warfarin (O4 in Fig. 1). The Phe108/304 gate is closed in this simulation, as shown in Fig. S12B. In the third membrane-bound simulation, warfarin is also far from Cpd I, and no hydrogen bond is formed between the substrate and the Arg212 sidechain. In this simulation, Arg212 adopts a conformation in which the side chain is pointing out of the active site. The Phe108/304 gate goes from being closed to open during this simulation. Hence, it appears that Arg212, and the Phe108-Phe304 gate, are important for warfarin binding in CYP3A4 in the membrane-bound model.

For the solubilized protein simulations with R-warfarin bound, similar observations were made to those for the membrane simulations regarding the distance of warfarin from Cpd I and the hydrogen bonding between warfarin and Arg212. The Phe108/304 gate remained closed during all of the simulations.

Differences between warfarin mobility within the active site were also observed. These appear to correlate with with the opening of the 2b channel, with increased motion of warfarin within the active site when this channel was open (Fig. S13). When the 2b channel is open, the distance between the Cpd I oxygen and warfarin is consistently greater than when the channel is closed.

#### Effect of substrate on heme propionates

The heme propionate groups in P450s contribute towards the binding of this prosthetic group to the protein. In a recent study, [14] a detailed comparison was made between the structural information known for soluble and membrane-bound P450s. It was found that the A propionate adopts two conformations in the known protein crystal structures: one in which the propionate is located on the proximal side of the heme (i.e. the same side as the coordinating cysteine), and one in which it is on the opposite distal side. The B propionate resides on the proximal side in all of the known crystal structures. In membrane-bound P450s, the A propionate was predominantly found to be on the distal side of the heme, and the converse was true of the soluble P450s. As mentioned in the Introduction, there are no known X-ray crystal structures of membrane-bound P450s in the presence of the membrane environment: only the structures of the solubilized forms of the enzymes have been determined to date.

Analysis of the dihedral angles relating to the position of the heme propionate substituents relative to the heme porphyrin ring during the atomistic MD simulations revealed that in the membrane bound simulations, the propionates spend significant proportions of time above and below the plane of the heme (see Fig. S14). In the solution model, the propionates sample both conformations in the apo simulations but remain in the ‘down’ position for the warfarin bound simulations. Analysis of the salt bridges between the propionates and the surrounding residues (Arg375, Arg105, Arg130, Arg440 and Trp126) shows that a greater variation in these salt bridge distances occurs in the substrate-free models, which corresponds with the propionates adopting the ‘up’ conformation. Hence, it appears that the presence of substrate in the active site promotes the propionates retaining the ‘down’ conformation observed in the crystal structure. Propionates have been suggested to have an involvement in electron transfer for Compound I in P450s and peroxidases. [41]

### QM/MM calculations

As mentioned in the introduction, QM/MM calculations have not been previously performed on membrane-bound proteins. In the following section, QM/MM calculations of the membrane-bound model of CYP3A4 have been performed. The effect of membrane-binding on the electronic structure of the active oxidizing species (Compound I) has been investigated, as well as the energy barrier to the hydrogen abstraction from R-warfarin by Compound I. Starting structures for QM/MM calculations were obtained from the atomistic MD simulations described above, and truncated using the protocol described in the Methods section.

#### Electronic structure of Compound I

Compound I is an iron-oxo porphyrin radical cation species that is commonly believed to be the active oxidising species in P450s. [20], [42] The electronic structure of Cpd I in several CYP isoforms has been calculated previously using similar methods to those described here, however, these calculations have only been performed for soluble models. [27] In the previous work it was concluded that there is no significant difference in electronic structure between the human isoforms studied (CYPs 2C9, 2D6 and 3A4), and a slight difference between the human isoforms and the bacterial P450*_cam_* isoform. This is the result of differences in hydrogen bonding between the cysteine sulfur coordinated to Cpd I and the backbone N–H of a neighbouring glycine. Structural differences between soluble and membrane-bound CYPs may affect the local environment surrounding the active site, leading to changes in Cpd I. Hence, the magnitude of this effect has been calculated here.

QM/MM minimizations were performed on structures obtained from each MD simulation, obtained at 5 ns intervals. As detailed above, particularly for the membrane-bound models, some initial relaxation of the protein occurs during the initial period of the simulation. Hence, minimizations were only performed on structures from the last 20 ns of each simulation. This yielded a total of 15 structures for each model. For the soluble models, each system was truncated such that all water molecules and ions that were at a distance of 5 Å or greater from the protein were deleted. The same truncation procedure was used for the membrane models, and the membrane was additionally removed. It is unlikely that the membrane will have any direct electrostatic effect on the QM region, due to the large separation between the heme and the membrane, and therefore it seems reasonable not to include this region in QM/MM calculations, in order to reduce the computational expense. It is important to note that when the majority of the protein is fixed during the QM/MM calculations, any structural effects of the membrane on the QM region will be retained.

**Figure 6:**
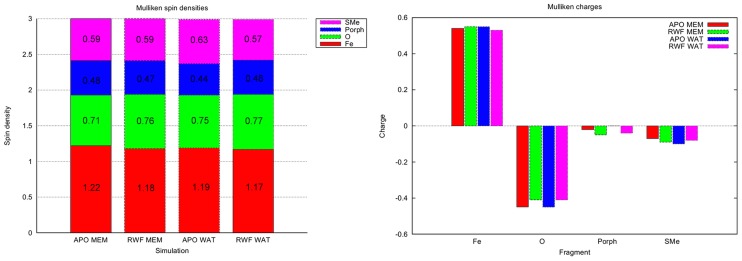
Average Mulliken spin densities (A) and charges (B) for fragments calculated from QM/MM energy minimizations. Calculated at the B3LYP-D:6-31G/CHARMM27 level of theory. M*_A_* and M*_R_* denote the membrane apo and R-warfarin bound models, respectively. S*_A_* and S*_R_* denote the solvated apo and R-warfarin bound models, respectively. Small differences are observed between the average values in the calculated properties. However, these differences are of similar order to the variation in values between different time points in a given simulation.

Mulliken spin densities and charges were calculated for the optimized structures, and the average values are shown in Fig. 6. The standard deviations of these quantities are low (0.10 or less) and are provided in Tables S2 and S3. These analyses have been shown previously to provide a reasonable indication of differences in electronic structure between different forms of Cpd I. [25], [27] The calculated values are in good agreement those calculated in previous work. [25], [27] Very little difference in the computed charges and spins of the individual fragments is observed between the membrane and soluble systems. This suggests that the presence of the membrane does not cause any structural effect in the region surrounding the heme that is sufficient to affect the electronic structure of Cpd I. The charges and spin densities on the ferryl oxygen are slightly larger for the substrate bound models (by ∼0.05) both in the presence and absence of membrane. This is consistent with previous work, [27] in which Cpd I was found to be more oxidising in the presence of substrate. This is believed to be due to the displacement of water (which can hydrogen bond to the Cpd I oxygen) by the substrate, and has been discussed in detail previously. [27]

The Fe–S bond length was also measured for the QM/MM optimized geometries containing R-warfarin, as this has been shown previously to vary with the distribution of the third unpaired electron between the sulfur lone pair and the porphyrin a_2*μ*_ orbital. [27] A longer Fe–S distance is usually accompanied by an increase in unpaired spin density on sulfur. The average values for the solution and membrane model are 2.622 Å and 2.697 Å respectively. The standard deviation of these values are 0.075 Å and 0.110 Å, respectively. The membrane-bound model calculations therefore contain a slightly higher Fe–S distance on average, however, this is not statistically significant, as the membrane average distance falls within the standard deviation of the solution simulations average value.

#### Hydrogen abstraction from R-warfarin

Aliphatic hydroxylation by P450s is widely-accepted to proceed via the H-abstraction/rebound mechanism. [43] The first (and rate-limiting) step is hydrogen-atom abstraction from the substrate by Cpd I, leading to a substrate radical. The ground state electronic structure of Cpd I is a mixture of near-degenerate quartet and doublet spin states. [44]–[46] In calculations of aliphatic hydroxylation of other substrates, it has been found that barriers to hydrogen abstraction are effectively the same for the doublet and quartet spin states of Cpd I. [47] Hence, in the current study, only the quartet spin state of Cpd I has been considered.

Five hydrogen abstraction profiles were calculated for the soluble and membrane-bound models. Starting structures were obtained from restrained atomistic MD simulations, where an harmonic restraint was applied to the distance between the ferryl oxygen atom of Cpd I and H10 of R-warfarin. The simulations produced structures that were close to the transition state of hydrogen abstraction, with an O–H distance of 1.8 Å. The reaction profile was generated by performing QM/MM minimizations with the O–H distance fixed to distances between 1.0 and 3.0 Å, in the forward and reverse directions, starting at 1.8 Å. The reaction barrier was taken as the difference in energy between the lowest and highest points on the potential energy surface, i.e. the reactant complex and transition state, respectively. As found in previous calculations of H-abstraction in CYPs, [8], [21], [24], [29]–[31] the transition states are located at an approximate O–H distance of 1.25 Å. The barriers calculated (with the larger basis set) for the soluble and membrane-bound models are provided in Table 2. The barriers calculated in the solution model range from 14.7 – 23.3 kcal/mol and the barriers calculated in the membrane model system range from 14.2 – 20.3 kcal/mol. It is common to observe a range of barriers when starting from different structures, and the lower barriers are more likely to be representative of the reactivity of the enzyme. [8], [21] This behaviour reflects the complex conformational nature of proteins. As detailed in our previous work, [21] it is convenient to apply a Boltzmann weighting factor when calculating the average barriers. Applying this averaging procedure to the calculated barriers yields a barrier of 15.0 and 15.6 kcal/mol for the membrane-bound and solublized models of CYP3A4, respectively. From these computed barriers, it is apparent that the barriers calculated from structures generated from the membrane-bound simulation of CYP3A4 do not differ significantly from those calculated from simulations performed in the absence of membrane and transmembrane helix.

**Table 2:** QM/MM activation energy barriers [in kcal/mol] for oxidation at C10 of R-warfarin.

Profile	Water	Membrane
p1	19.3	20.3
p2	15.9	15.0
p3	23.3	18.8
p4	20.1	14.2
p5	14.7	17.8
Ave	18.7	17.2
Boltzmann-weighted Ave	15.6	15.0
St. Dev.	3.4	2.6

Calculated at the B3LYP-D:6-311++G(d,p)//B3LYP-D:6-31G/CHARMM27 level. Calculated for the quartet spin state of Cpd I.

**Figure 7:**
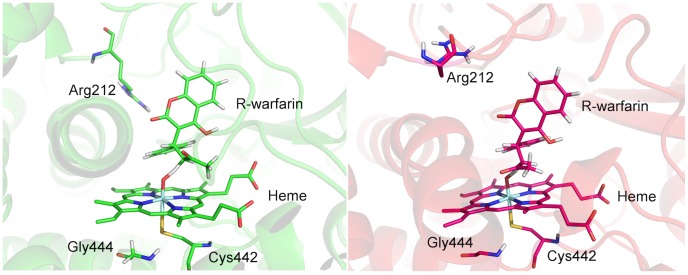
Transition state geometries for hydrogen abstraction pathways with lowest barrier. Left: profile calculated from membrane simulation; right: profile calculated from water simulation. Calculated at B3LYP-D:6-31G/CHARMM27 level of theory. Arg212 shown explicitly and has been proposed to play a role in substrate specificity of CYP3A4. In the lowest-energy profiles generated it is observed to hydrogen bond with the O2 atom of R-warfarin in the membrane-bound simulation but not in the soluble case where Arg212 shows increased flexibility.

The transition state geometries for the lowest energy pathways are displayed in Fig. 7. The orientation of warfarin in the two structures is very similar. A notable difference between the membrane and solution transition state structures is the conformation of the Arg212 side chain. In the solubilized protein model, the Arg212 side chain points away from the active site, but in the membrane-bound model, the side chain points towards the substrate, forming a salt bridge with O2 of warfarin. Arg212 has been identified as important in the binding of substrates, [39], [48] and therefore it is surprising that this interaction is missing in the lowest energy pathway for the solution model, but is present in the membrane-bound model. A further difference between the two models is the conformation of the propionate side chains of the heme group. In the transition state structure derived from the membrane-bound model, the propionates lie above the plane of the heme, but in the solubilized model the propionates are below the heme plane (as is the case in the 1TQN crystal structure). Further investigation will be required to examine whether these differences are due to the membrane environment.

Docking and QM studies of the N-dealkylation of 4-aminopiperidines by CYP3A4 indicated that Ser119 plays an important role in the binding the substrate in a position to aid reaction. [49] However, in the current work no interactions were observed between R-warfarin and this residue.

In both of the transition state geometries shown in Fig. 7 there is a hydrogen bond formed between the backbone NH group of Gly444 and the sulfur of Cys442. It has been shown in previous work [27] that this interaction has a significant influence on Cpd I. In the absence of this hydrogen bond, the Fe–S bond length elongates and results in higher barriers to hydrogen abstraction. It is apparent from this study that the H-bonding environment surrounding the Cys442 sulfur is not affected by the presence/absence of the membrane. Therefore, the electronic structure and reactivity of Cpd I is also unaffected by the presence of the enzyme binding to the membrane.

## Discussion

Multiscale modelling is recognised as a central goal in molecular simulation of complex biological systems. [50] Efficient and practical protocols and methods for realistic multiscale modelling have the potential to contribute to many important problems in biology. Here, we have demonstrated a practical multiscale simulation approach in an application to a key system involved in drug metabolism.

CGMD simulations provided an unbiased method to generate an equilibrated model of the membrane-bound CYP3A4. A significant degree of distortion of the lipid bilayer was observed in the region in contact with the protein. Thus it is clear that the planar model representation of the lipid bilayer used in the OPM method is something of a simplification. Consideration of the non-planar properties of the membrane is expected to play a role in the mode of substrate interaction with the protein.

The CG model of a protein:membrane complex was successfully converted to a atomistic model, using an automated procedure that is applicable to other proteins and is compatible with other forcefields. A stable protein:membrane complex was obtained, in which the location and orientation of the protein in the lipid bilayer stabilized within a relatively short simulation timescale. Comparison of the atomistic simulations of the membrane-bound model with the corresponding solubilized form of the enzyme showed that the binding of the enzyme to the membrane has a limited effect on the overall protein flexibility. However, significant differences are observed in the behaviour of the reactant and product channels between the membrane-bound and solution models. This agrees well with previous multiscale simulations of CYP2C9. [19]

The atomistic simulations of CYP3A4 with R-warfarin revealed interactions that appear important to substrate-binding. Arg212 was found to interact with the substrate, and the gate formed by the sidechains of Phe108 and Phe304 were found to influence the orientation of warfarin in the active site. Additionally, the opening and closing of the 2b access channel was found to influence the mobility of warfarin in the active site.

The effect of membrane-binding on the residues surrounding the proximal side of the heme has been found to be minimal and, as a result of this, the local electrostatic environment surrounding the QM region is effectively the same in the membrane-bound and solution simulations. Hence, the electronic structure of Cpd I is not affected by the presence of the membrane, nor are the barriers to hydrogen abstraction. The finding is important, because it supports the use of engineered P450s as models of their membrane-bound counterparts. Further studies could investigate the roles of different lipids on activity.

Human drug-metabolizing P450s, including CYP3A4, have been identified as being responsible for adverse drug reactions due to drug-drug interactions. One possible cause of drug-drug interactions is competitive binding of substrates to P450 isoforms. Multiple substrate binding to CYP3A4 has been studied both experimentally [51]–[53] and theoretically [54], [55]. The large active site cavity of CYP3A4 can easily accommodate more than one substrate molecule simultaneously. Whilst we have not studied this phenomenon in detail here, a calculation was performed in which a second molecule of R-warfarin was docked into the active site of CYP3A4 when a first R-warfarin molecule was already present (see Fig. S15). We observed from this preliminary docking calculation that more than one molecule of R-warfarin can indeed be accommodated at any one time.

The multiscale approach outlined here should be useful in future studies to investigate the entire reaction cycle of CYPs. Firstly, the entrance of substrate into the active site through the membrane may be simulated. This process is perhaps best suited to accelerated simulations methods e.g. a metadynamics approach, [56] because (for models of this size) such an event is beyond the timescale of atomistic MD simulations at present. The steps leading to the formation of Cpd I and hydroxylation of the substrate could be modelled with QM/MM. Finally, the exit of the product may also be investigated, following a conversion back to a CG model following the QM/MM calculations. As larger simulations become more computationally feasible, it will be possible to include more components, e.g. including the reductase protein in the simulation, however, this will require further evidence for orientations.

QM/MM calculations may also be used to refine CG and atomistic MD parameters, particularly those used for substrates. Empirical valence bond parameters [57] for this reaction might also be calculated, fitted to the QM/MM calculated barriers, enabling free energy barriers to be calculated, and the effects of point mutations on the barrier to be investigated.

## Methods

**Figure 8:**
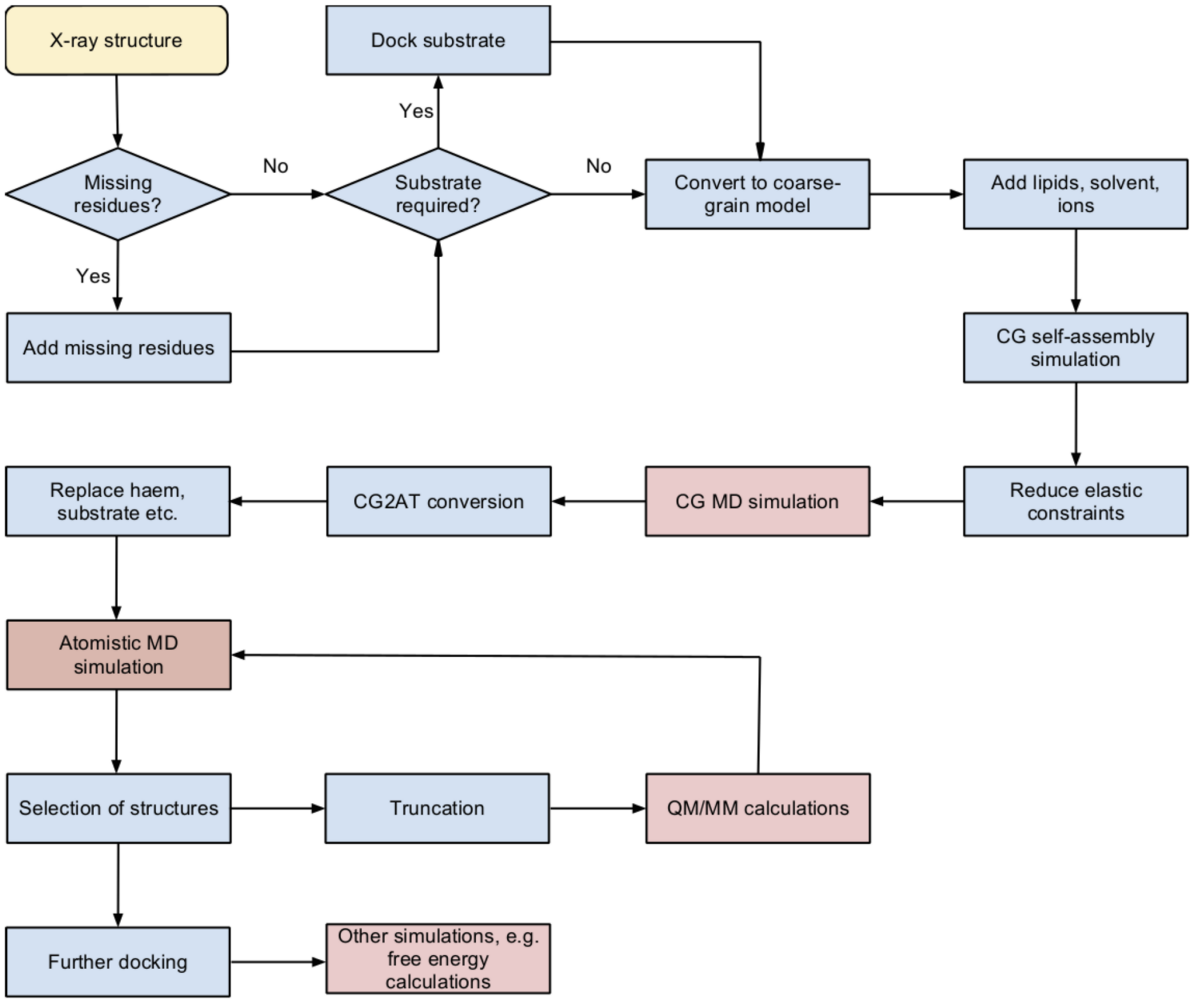
Flow diagram for preparation of protein:membrane model for QM/MM calculations. Blue stages correspond to preparation steps whereas pink stages involve molecular dynamics (MD) simulations and QM/MM calculations. In the initial step (yellow) the structure of the protein of interest is downloaded from the PDB. The following steps describe the unbiased coarse grained (CG) set up of a protein in the presence of a chosen lipid composition. Following these preparatory stages CGMD simulations are run to allow the bilayer to self-assemble in an unbiased manner around the protein. This CGMD protein-membrane simulation box may be converted to atomistic resolution and atomistic MD simulations performed to look at conformational changes. The outcome of these simulations may then be used as the basis of QM/MM calculations or other calculations of interest.

The protocol for building a protein:membrane model is outlined in Fig. 8. The process starts with the selection of an X-ray crystal structure. The 1TQN crystal structure was used in this study. [12] Missing residues were added (residues 282-285 and the transmembrane helix) by homology modelling using the MODELLER program [58]. For the simulation models containing substrate, R-warfarin was docked into the active site using AUTODOCK VINA. [59] The Arg212 side chain, which has been identified as important in ligand binding in CYP3A4, points into the active site in the 1TQN structure. This is in contrast to the 1W0G structure, [40] where the residue points away from the heme. Hence, this residue was treated as flexible during the docking calculations. Nine docking poses were located during the docking calculation. All of these placed the substrate in the active site and possessed similar calculated binding affinites, ranging between −9.0 and −8.5 kcal/mol. The docking pose in which C10 was located closest to the heme Fe (pose 8, see Fig. S16) was selected for simulation. Each model was converted into a course-grained model using the *make_cg_martini.pl* script, which prepares input for a GROMACS simulation using the MARTINI 2.1 CG forcefield. [60]–[63] Lipids were added to the simulation box in random orientation using the *genbox* utility in GROMACS, according the composition present in the natural environment of the enzyme. In the present study, 1-palmitoyl-2-oleoyl-sn-glycero-3-phosphocholine (POPC) and 1-palmitoyl-2-oleoyl-sn-glycero-3-phosphoethanolamine (POPE) were used in an approximated 3:1 ratio. These are the main components of the endoplasmic reticulum membrane, according to the OPM database. [15] CG water beads and ions ([NaCl]  =  0.15 M, excess charge neutralized) were also added to the simulation box at this stage. 5000 steps of steepest descent energy minimization are performed before the initial CG self-assembly simulation. The CG simulations were performed at 310 K and 1 atm, using Berendsen temperature and pressure coupling (semiisotropic). A timestep of 20 fs was used and each simulation was 1 *μ*s in length. During the CG simulation, the lipid bilayer forms around the transmembrane helix. Usually in a CG simulation, elastic constraints are applied to the protein beads, in order to preserve the tertiary structure of the protein. Because the exact orientation of the transmembrane helix relative to the rest of protein is unknown, it was necessary to relax some of the elastic constraints, particularly around the region where the transmembrane helix is attached to the protein. CG simulations were performed using GROMACS 4.6. [64]–[67] An additional CG simulation was then performed to sample different conformations around this region of the protein. Once a suitable protein:membrane complex was obtained, the conversion from CG model to atomistic resolution was performed using the previously defined CG2AT protocol. [2] During this protocol, the lipids are first converted from a CG to AT representation using a fragment library based approach, and undergo energy minimisation, before the protein undergoes a similar transformation. The substrate was not present during the CG simulations and was introduced to the atomistic representation of the protein by superposition with the initial docked pose prior the conversion to CG. The atomistic model generated from the previous step was then aligned during conversion (automated within the *cg2at* script).

Atomistic MD simulations were performed using NAMD version 2.9. [68] The CHARMM27 force field with CMAP corrections was used for protein atoms. [69], [70] The CHARMM36 force field [71] was used for lipids and the CHARMM implementation of the TIP3P model used for water (with Lennard-Jones parameters for the hydrogen atoms). [72] Parameters for Cpd I and warfarin were obtained from previous work. [8], [25] The structures obtained from the CG2AT conversion were energy minimized using 6000 steps of conjugate gradient minimization for all hydrogen atoms, followed by 6000 steps for all hydrogen and solvent atoms. The water molecules were then heated to 300 K and equilibrated for 20 ps in an NVT ensemble. The positions of all atoms were then energy minimized by 10000 steps of CG minimization. The system was heated to 300 K over 20 ps before equilibration of 1000 ps with constant temperature, pressure and area in the xy plane. It was necessary to include this extra step in order to maintain the shape of the membrane. The Nose-Hoover piston [73] and Langevin temperature control [74] were used during equilibration. Three production MD simulations of 50 ns were obtained for each system, started from heating stages with different initial velocities. The production MD simulations were run in the absence of a constant area constraint. A time step of 2 fs was used and bonds to hydrogen atoms were constrained with the SHAKE algorithm. The solution MD systems were solvated using the SOLVATE plugin in VMD. [75] During initial calculations the protein was solvated such that there was 12 Å of water surrounding the entire protein. Due to the shape of the protein it was found that this led to the protein interacting with its periodic image. Hence, the soluble simulations were repeated in a cubic box of water with the length of the longest side from the initial calculations (see Table S1 for box dimensions).

Convergence is a vital issue in MD simulations, which is why several independent CGMD simulations and 3 repeats of each 50 ns atomistic simulation were performed. This is generally regarded as better than running a single longer simulation. [76] It is difficult to prove convergence, but important aspects do need to be converged, e.g. the independent CGMD simulations led to a converged orientation of the protein within the bilayer. The properties that we have focused on in this work are the dynamic properties of the substrate channels, the reorganisation of protein near the protein-lipid interface, the active site volume and the behaviour of Arg212. By using data from multiple independent simulations, we are able to observe variation in these properties, within the timescales simulated, that are consistent with previous computational studies. We hence decided only to analyze the portions of the atomistic simulations where (in the case of the membrane-bound models) the orientation of the protein in the membrane ceased to vary to an appreciable extent (and was closest to the experimentally determined value). For this reason, only the last 20 ns of each atomistic simulation was used for analysis purposes.

QM/MM calculations were performed using the QoMMMa program. [77] The QM part of the calculations was performed in Jaguar version 5.5. [78] The QM region consisted of the substrate (where applicable), iron-oxo porphyrin (without heme substituents) and the proximal cysteine (modelled as SCH_2_). Valences of atoms at the QM/MM boundary were satisfied by using the link atom approach. [79] The B3LYP density functional was used [80]–[82] along with the LACVP basis set for iron [83] and 6–31G for all other atoms. [84] The quartet spin state of Cpd I was modelled with a restricted open-shell approach. An empirical dispersion correction [85] was applied in all QM calculations, as this was found to be important in previous work. [29,29] For consistency with the atomistic MD simulations, the CHARMM27 force field [69], [70] was used for the MM part of the QM/MM calculations using Tinker version 6.1. Structures for QM/MM calculations of the electronic structure of Cpd I were obtained from the last 20 ns of each MD unrestrained MD simulation at 5 ns intervals. Structures for reaction profiles were obtained from restrained MD simulations that were performed as follows. A structure was selected from the unrestrained simulations in which the distance between the Cpd I oxygen and C10 was at the minimum distance. A restrained energy minimization (10000 steps of CG) was then performed on this structure, with an harmonic restraint centred on 1.2 Å applied to the O-H5 distance (corresponding to the Cpd I oxygen and hydrogen attached to C10) with a force constant of 100 kcal/mol/Å^2^. The system was then heated to 300 K over 20 ps and equilibrated for 180 ps with the same restraint present. A production MD simulation was then performed of 10 ns, again with the same restraint on the O-H distance. Structures were truncated prior to QM/MM calculation such that all water molecules and ions at a distance greater than 5 Å of any protein atom were removed. In the membrane-bound structures, the membrane was excluded from the QM/MM calculations. All residues that contained at least one atom that was within 5 Å of any atom of Cpd I or substrate were included in the QM/MM energy minimization, while all other atoms were fixed to their initial positions.

Reaction energy profiles were generated by performing QM/MM energy minimization with a restraint corresponding to the O-H5 distance (force constant 1000 kcal/mol/Å^2^). Structures were first optimized with a reaction coordinate value of 1.8 Å and then in increasing and decreasing intervals of 0.1 Å to 3.1 and 0.9 Å. This approach has been used previously for modelling the hydroxylation of S-warfarin in CYP2C9. [8]

All of the scripts used in this work are available to download at http://ccpforge.cse.rl.ac.uk/

## Supporting Information

Figure S1 **Elastic network used to maintain secondary and tertiary structure in coarse-grained molecular dynamics simulations.** The protein backbone is shown as a blue trace. Elastic network restraints are shown in red. Backbone atoms within a 7 Å cut-off of other backbone particles have a restraint applied. The dashed black lines represent the elastic network bonds within this cut-off that have been removed to permit motion of the modelled transmembrane helix compared to the globular domain.
(TIFF)

Figure S2 **Interactions between cytochrome P450 3A4 and the hydrophobic region of the membrane.** The protein is shown as a backbone trace and coloured according to interactions with the alkyl tails of the lipids (Blue  =  0% simulation in contact, red  =  100% simulation in contact). The A-anchor is the part of the globular domain forming the most hydrophobic interactions, followed by the G′ helix and F′-helix.
(TIF)

Figure S3 **Comparison between orientation of CYP3A4 calculated using CGMD simulations (surface) and orientation predicted by the Orientation of Proteins in Membranes (OPM) database (red spheres).** The average position of the phosphate particles of the lipid headgroups in the reference frame of the protein (calculated and coloured as according to main text Figure 2) are shown as a surface. The transparent red spheres are the position of the membrane downloaded from the OPM database, which generates membrane protein orientations based on experimental data. There is a clear deviation in the angle of the membrane normal relative to the protein between the two models. However, the local deformation of the lipid bilayer in the region of the protein that occurs in molecular simulations leads to the two membrane lipid headgroup positions aligning in this region.
(TIF)

Figure S4 **Secondary structure analysis for atomistic MD simulations of (a) membrane-bound apo; (b) membrane-bound warfarin-containing; (c) solubilized apo and (d) solubilized warfarin-containing CYP3A4.**
(TIF)

Figure S5 **Conformational clustering of simulations.** Each of the simulations was conformationally clustered based on the backbone particles of the entire globular domain (left) and the 141 residues bordering the active site (right). Top representative frames were generated using NMRClust. The lowest RMSDs are between the soluble simulations with and without substrate bound. The maximum active site RMSD between all simulations is 2.4 Å.
(TIF)

Figure S6 **Top, middle: Root mean square fluctuation [in nm] of protein backbone C** ***α*** **atoms from average structure for membrane (mem) and solution (wat) simulations, respectively.** Bottom: B factor [in nm^2^] for C*α* atoms in 1TQN crystal structure.
(TIF)

Figure S7 **Active site volumes of each atomistic MD simulation.** Images were generated from the frame at 40 ns using HOLLOW. Approximate active site volumes calculated over the final 20 ns of each trajectory are shown with the standard deviation in parentheses.
(TIF)

Figure S8 **CAVER tunnels for (a) apo mem (b) rwf mem (c) apo wat (d) rwf wat.** Computed at 5 ns intervals for the last 20 ns of each trajectory.
(TIF)

Figure S9 **Top 6 ranked CAVER tunnel clusters for (a) apo mem (b) rwf mem (c) apo wat (d) rwf wat.** Computed at 5 ns intervals for the last 20 ns of each trajectory.
(TIF)

Figure S10 **Position of substrate ingress channels relative to the membrane.** The top 6 channels calculated from CAVER are shown for the membrane-apo simulations and overlaid with the average positions of the membrane headgroups over the simulations. The 3 channel (coloured green) between the F′- and G′-helices is directed into the membrane.
(TIF)

Figure S11 **RMSD of warfarin, calculated relative to starting position (t = 0).** w1, w2, w3 refer to soluble simulations 1, 2 and 3 respectively. m1, m2, m3, refer to membrane simulations 1, 2 and 3, respectively.
(TIF)

Figure S12 **Structural snapshots from atomistic simulations of R-warfarin in the membrane-bound model of CYP3A4.** Arg212 is shown in green and the gating residues Phe108 and Phe104 are displayed in purple. A. Structure from simulation 1 (after 30 ns). Arg212 forms a hydrogen bond to O2 of warfarin. The Phe108-Phe304 gate is open during this simulation. B. Structure from simulation 2 (after 45 ns). Arg212 forms a hydrogen bond to O4 of warfarin. The Phe108-Phe304 gate is closed during this simulation.
(TIF)

Figure S13 **Correlation of warfarin motion gate 2b opening.** The motion of warfarin (calculated in terms of the variation of the distance between the iron oxygen and the centre of mass of warfarin) within the active site increases with 2b gate opening. No correlation with other gates was found.
(TIF)

Figure S14 **Heme propionate A dihedral angle (C3A-C2A-CAA-CBA) for all atomistic MD simulations.** The dihedral angle value of +100° corresponds to the propionate being located on the distal side of the heme, a value of −100° corresponds to the propionate being in the proximal position.
(TIF)

Figure S15 **Docking result for multiple substrate binding with R-warfarin in CYP3A4.** AUTODOCK VINA docking calculations were repeated using the R-warfarin-docked protein as the receptor input, and a second warfarin molecule as the ligand. The lowest-energy docked pose from this calculation places the second R-warfarin in the active site of CYP3A4. The second R-warfarin is shown in light grey. The atomistic model of the initial membrane-bound protein with a single R-warfarin molecule docked is shown in green. The heme group, Arg212, and Cys442 are shown in stick representation as in Figure 7.
(TIF)

Figure S16 **Docking pose selected as starting point for MD simulations of CYP3A4 with R-warfarin.** Calculated using AUTODOCK VINA with the 1TQN crystal structure. The Arg212 residue (shown) was treated as flexible, the rest of the protein was treated as fixed. Nine docking poses were found, pose 8 (shown) was found to have the smallest distance between C10 (the position to undergo hydroxylation) and the heme iron. The calculated binding affinity of this pose is −8.5 kcal/mol.
(TIFF)

Table S1 **Mean (and standard deviation) root mean squared deviations [in Å] of positions of protein backbone alpha carbon atoms from initial structure during entire MD simulations.** Box dimensions for the production runs are shown in Å, as well as the total number of water molecules present in each simulation set-up.
(DOCX)

Table S2 **Standard deviations of the values of the Mulliken atomic spin densities computed for the membrane-bound and solubilized CYP3A4 with R-warfarin bound (MR and SR, respectively) and without warfarin bound (MA and SA, respectively) optimized at the B3LYP-D:6-31G/CHARMM27 level of theory (average values are displayed in** **Figure 6A** **).** Calculated for 15 structures optimized at 5ns intervals over the last 20 ns of three 50 ns atomistic MD simulations.
(DOCX)

Table S3 **Average values of the Mulliken atomic charges computed for the membrane-bound and solubilized CYP3A4 with R-warfarin bound (MR and SR, respectively) and without warfarin bound (MA and SA, respectively) optimized at the B3LYP-D:6-31G/CHARMM27 level of theory (average values are displayed in** **Figure 6A** **).** Calculated for 15 structures optimized at 5ns intervals over the last 20 ns of three 50 ns atomistic MD simulations. Standard deviations are given in parentheses.
(DOCX)

Table S4 **Percentage of gates that are open during atomistic MD simulations of membrane-bound (MR and MA) and solubilized (SR and SA) CYP3A4.** The subscript labels R and A corresponds to the simulation models including and excluding R-warfarin, respectively. A gate is defined as open if the distance between the centre of mass of the two gating residue side chains is less than 7 Å.
(DOCX)
