# Supplementary figures and images for: A Multiscale Approach to Modelling Drug Metabolism by Membrane-Bound Cytochrome P450 Enzymes
Source: PLoS Comput Biol. 2014 Jul 17;10(7):e1003714. doi: 10.1371/journal.pcbi.1003714 (PMC4102395; doi:10.1371/journal.pcbi.1003714)

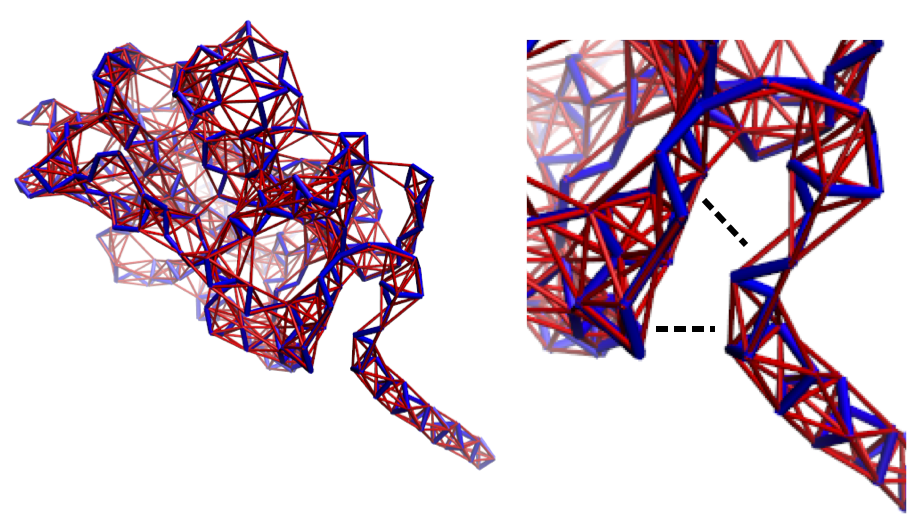

Supplement: Figure S1 — Elastic network used to maintain secondary and tertiary structure in coarse-grained molecular dynamics simulations. The protein backbone is shown as a blue trace. Elastic network restraints are shown in red. Backbone atoms within a 7 Å cut-off of other backbone particles have a restraint applied. The dashed black lines represent the elastic network bonds within this cut-off that have been removed to permit motion of the modelled transmembrane helix compared to the globular domain. (TIFF) [file pcbi.1003714.s001.tiff]

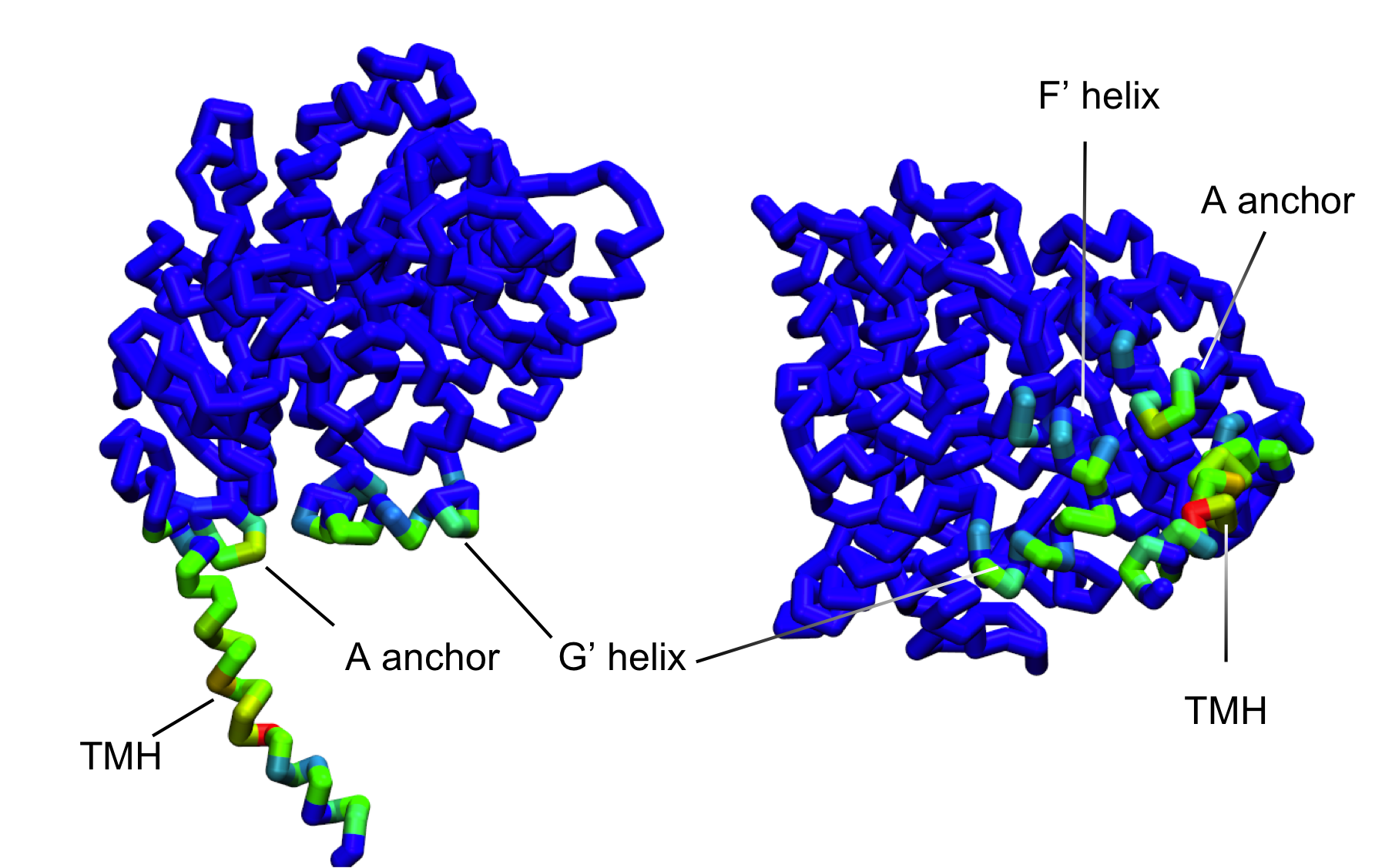

Supplement: Figure S2 — Interactions between cytochrome P450 3A4 and the hydrophobic region of the membrane. The protein is shown as a backbone trace and coloured according to interactions with the alkyl tails of the lipids (Blue = 0% simulation in contact, red = 100% simulation in contact). The A-anchor is the part of the globular domain forming the most hydrophobic interactions, followed by the G′ helix and F′-helix. (TIF) [file pcbi.1003714.s002.tif]

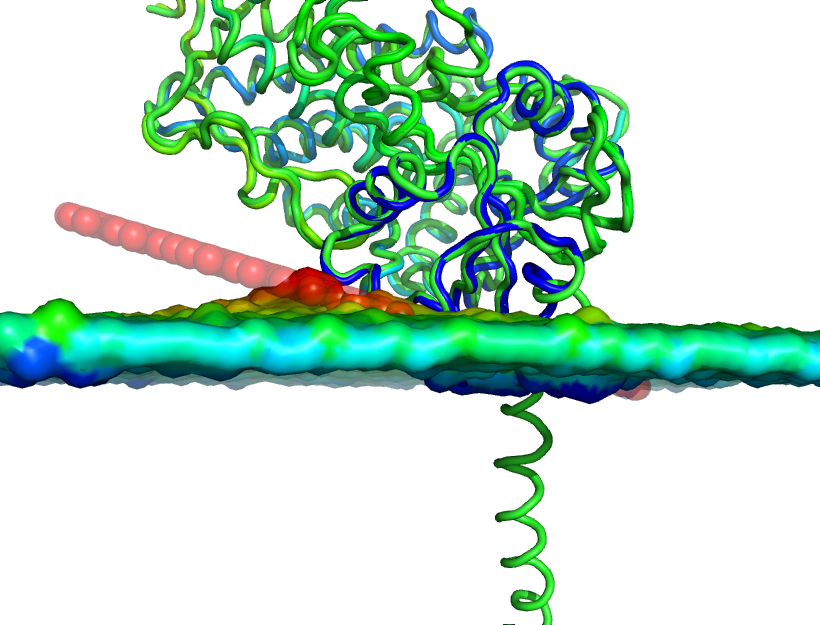

Supplement: Figure S3 — Comparison between orientation of CYP3A4 calculated using CGMD simulations (surface) and orientation predicted by the Orientation of Proteins in Membranes (OPM) database (red spheres). The average position of the phosphate particles of the lipid headgroups in the reference frame of the protein (calculated and coloured as according to main text Figure 2) are shown as a surface. The transparent red spheres are the position of the membrane downloaded from the OPM database, which generates membrane protein orientations based on experimental data. There is a clear deviation in the angle of the membrane normal relative to the protein between the two models. However, the local deformation of the lipid bilayer in the region of the protein that occurs in molecular simulations leads to the two membrane lipid headgroup positions aligning in this region. (TIF) [file pcbi.1003714.s003.tif]

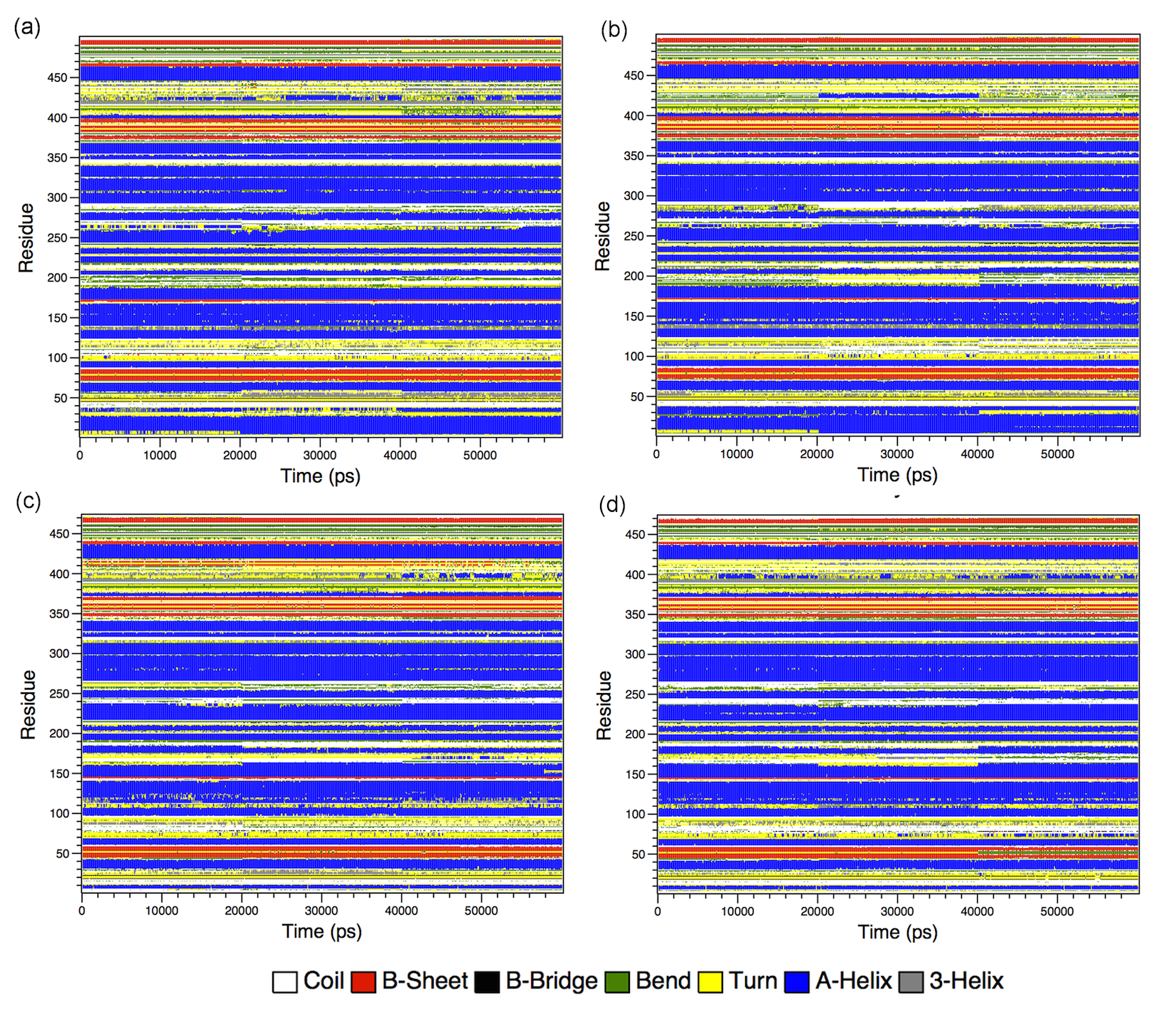

Supplement: Figure S4 — Secondary structure analysis for atomistic MD simulations of (a) membrane-bound apo; (b) membrane-bound warfarin-containing; (c) solubilized apo and (d) solubilized warfarin-containing CYP3A4. (TIF) [file pcbi.1003714.s004.tif]

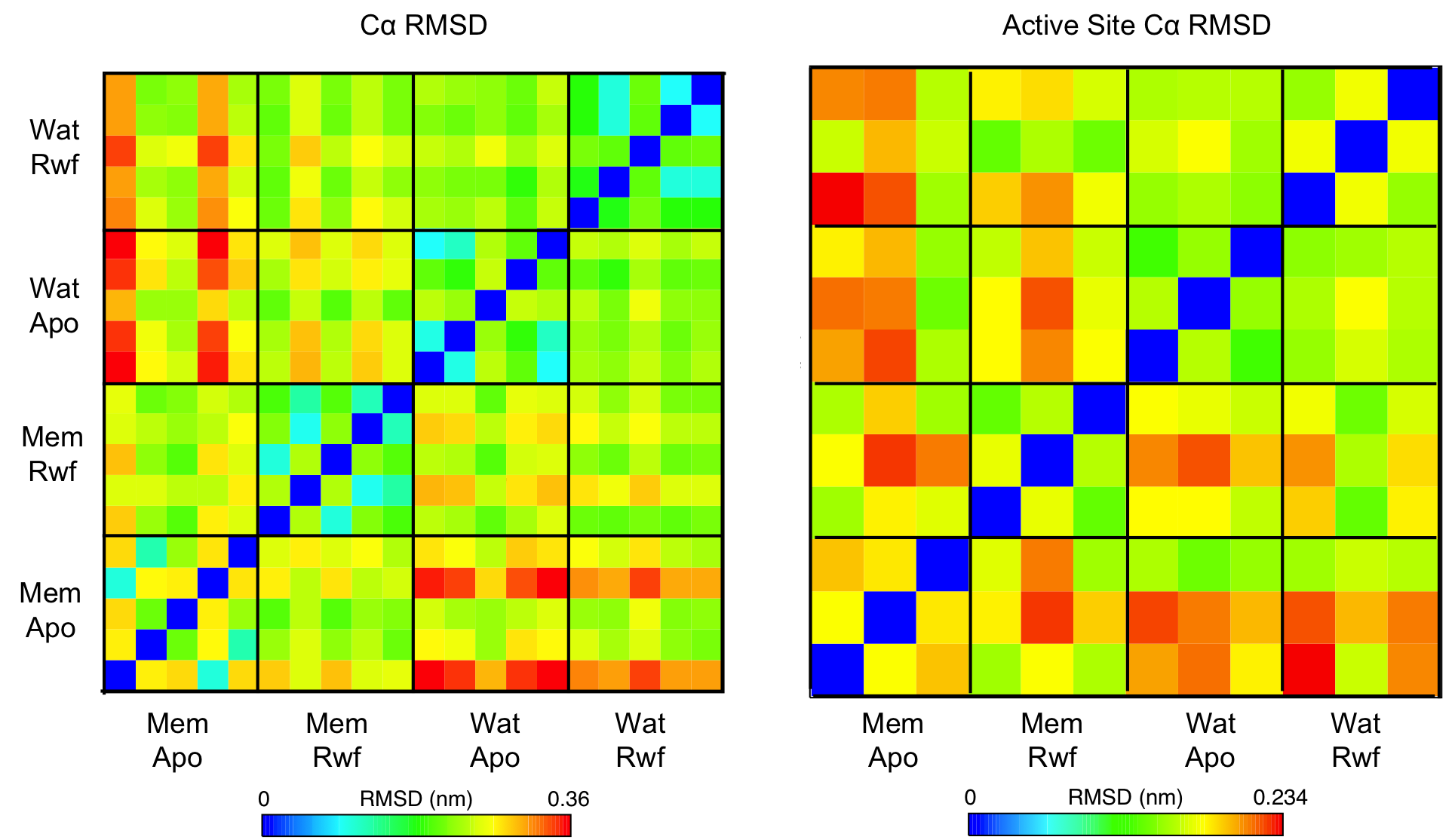

Supplement: Figure S5 — Conformational clustering of simulations. Each of the simulations was conformationally clustered based on the backbone particles of the entire globular domain (left) and the 141 residues bordering the active site (right). Top representative frames were generated using NMRClust. The lowest RMSDs are between the soluble simulations with and without substrate bound. The maximum active site RMSD between all simulations is 2.4 Å. (TIF) [file pcbi.1003714.s005.tif]

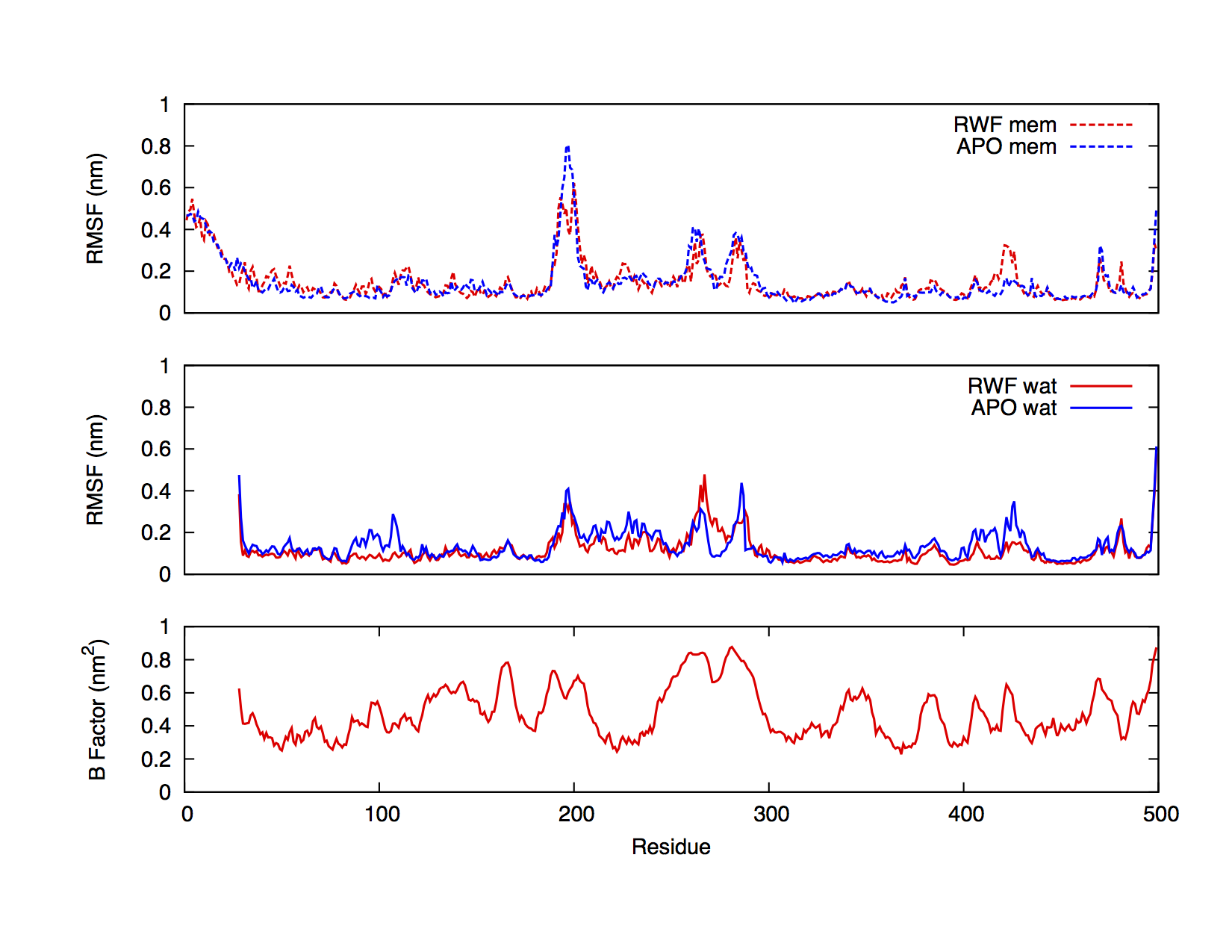

Supplement: Figure S6 — Top, middle: Root mean square fluctuation [in nm] of protein backbone C α atoms from average structure for membrane (mem) and solution (wat) simulations, respectively. Bottom: B factor [in nm2] for Cα atoms in 1TQN crystal structure. (TIF) [file pcbi.1003714.s006.tif]

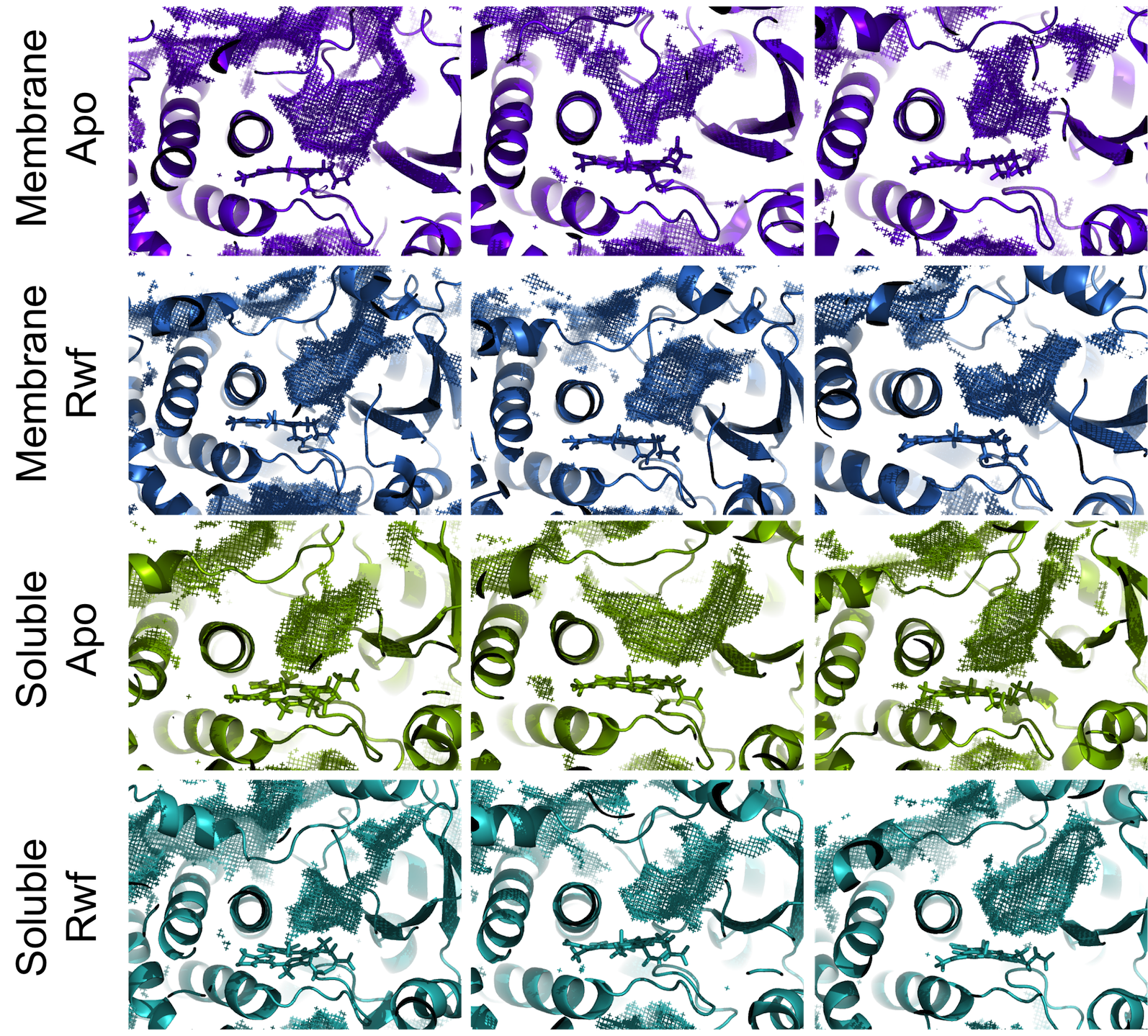

Supplement: Figure S7 — Active site volumes of each atomistic MD simulation. Images were generated from the frame at 40 ns using HOLLOW. Approximate active site volumes calculated over the final 20 ns of each trajectory are shown with the standard deviation in parentheses. (TIF) [file pcbi.1003714.s007.tif]

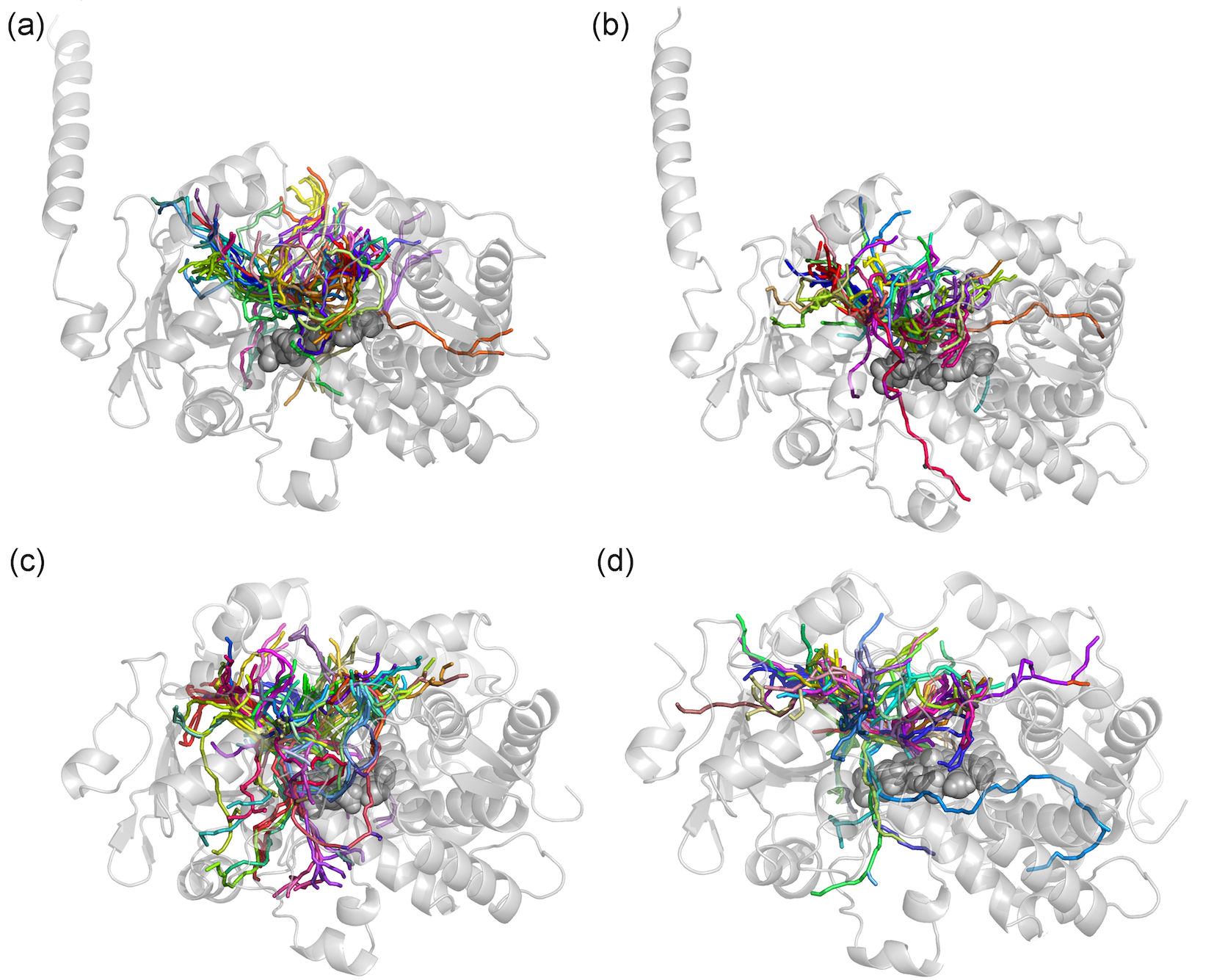

Supplement: Figure S8 — CAVER tunnels for (a) apo mem (b) rwf mem (c) apo wat (d) rwf wat. Computed at 5 ns intervals for the last 20 ns of each trajectory. (TIF) [file pcbi.1003714.s008.tif]

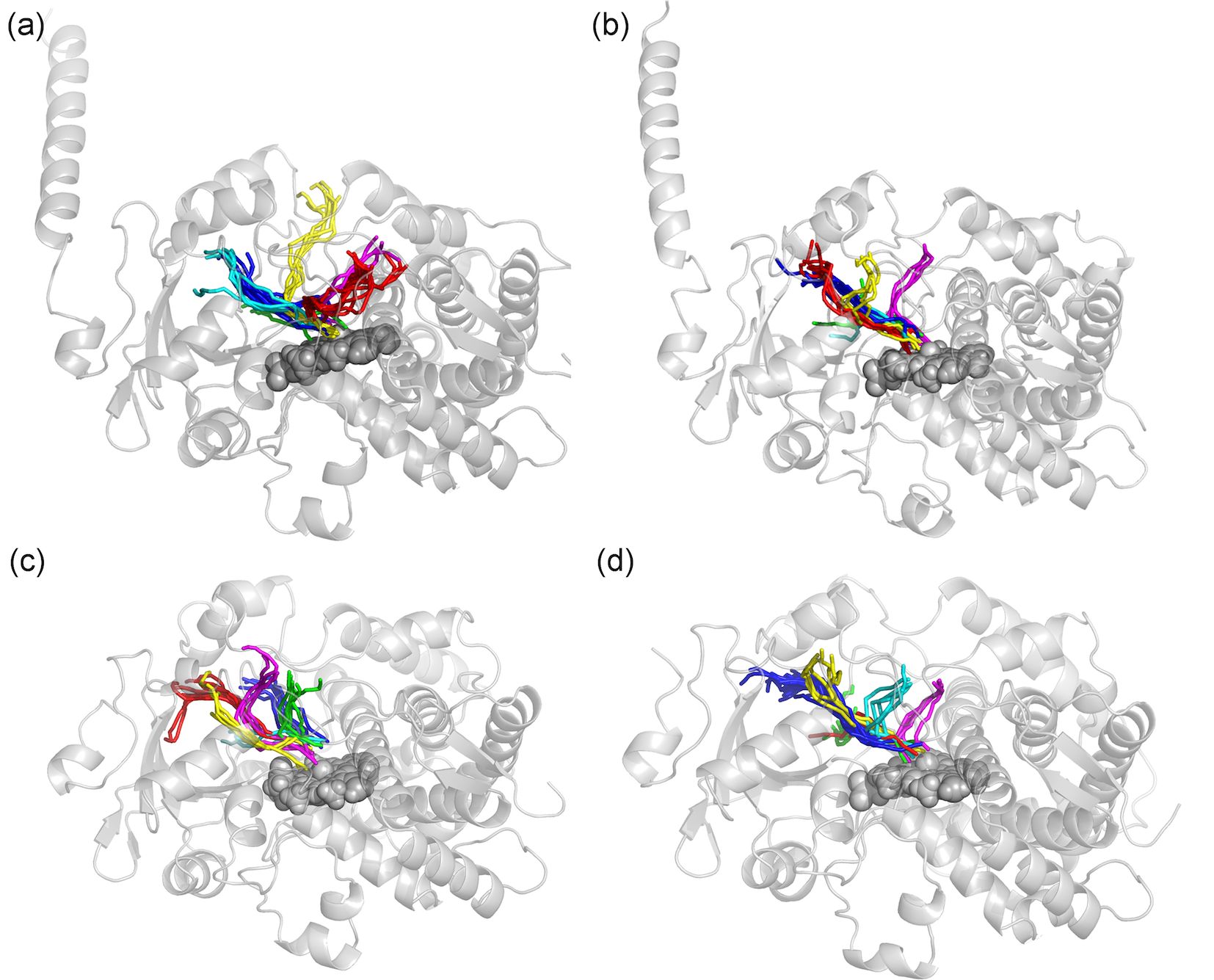

Supplement: Figure S9 — Top 6 ranked CAVER tunnel clusters for (a) apo mem (b) rwf mem (c) apo wat (d) rwf wat. Computed at 5 ns intervals for the last 20 ns of each trajectory. (TIF) [file pcbi.1003714.s009.tif]

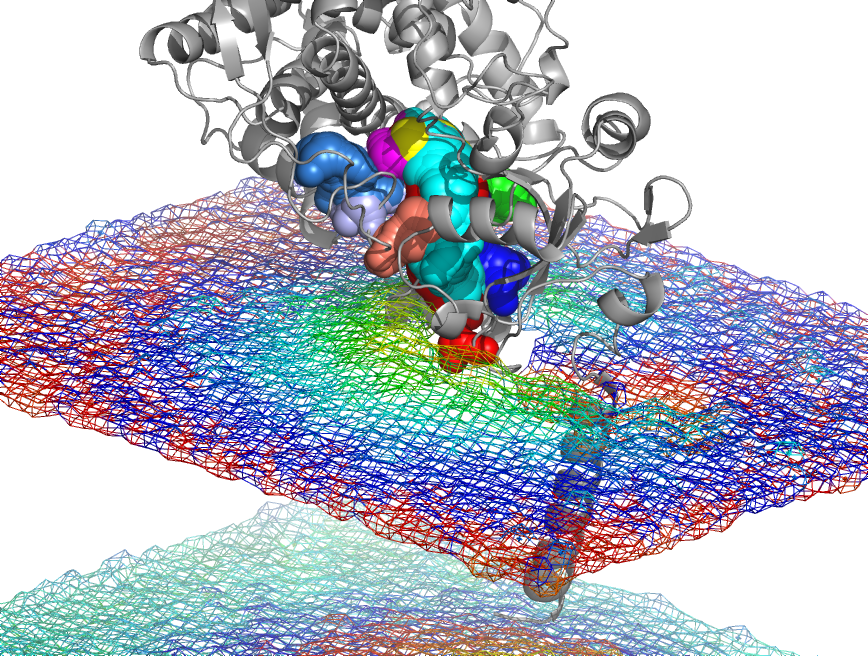

Supplement: Figure S10 — Position of substrate ingress channels relative to the membrane. The top 6 channels calculated from CAVER are shown for the membrane-apo simulations and overlaid with the average positions of the membrane headgroups over the simulations. The 3 channel (coloured green) between the F′- and G′-helices is directed into the membrane. (TIF) [file pcbi.1003714.s010.tif]

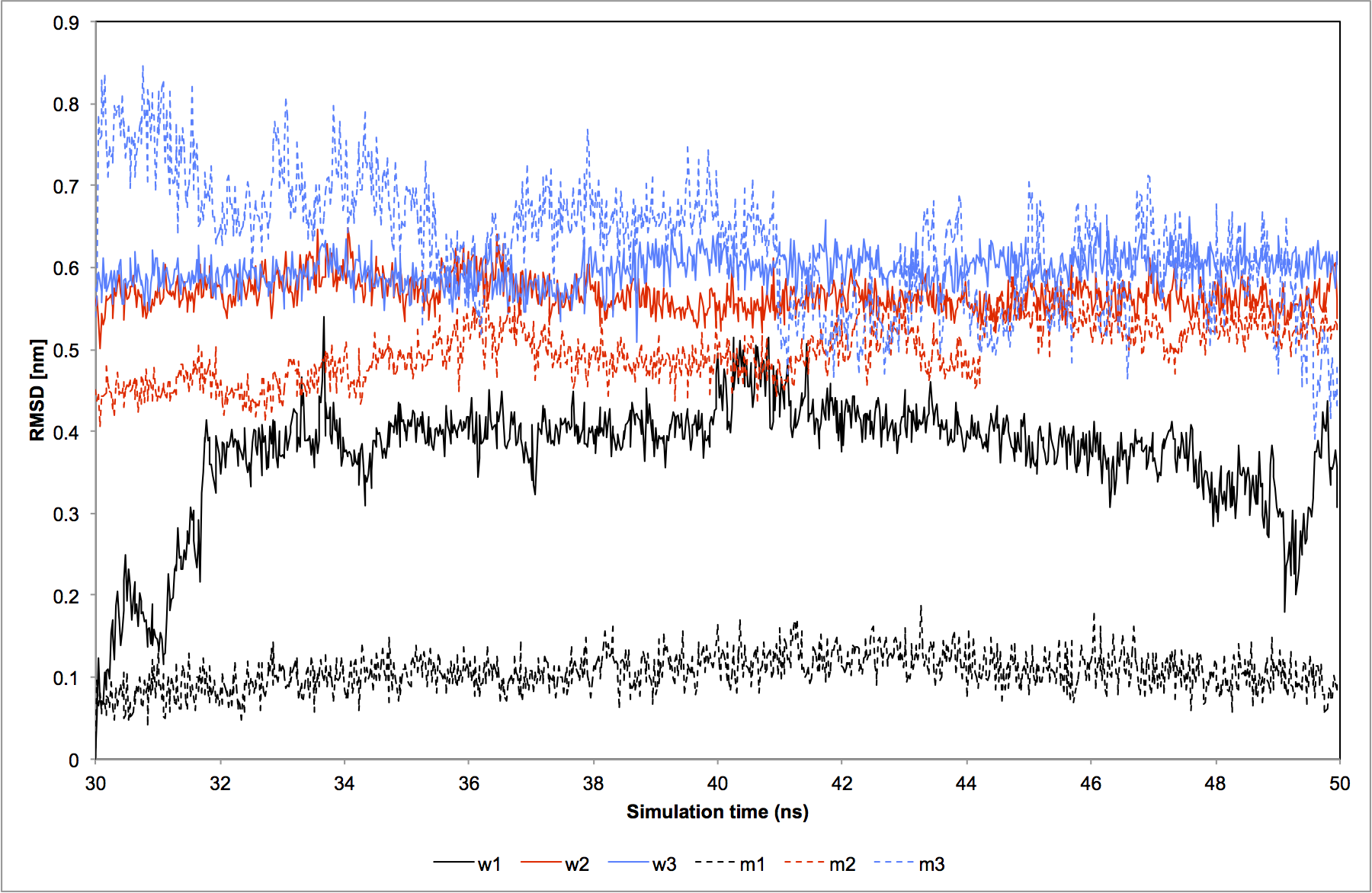

Supplement: Figure S11 — RMSD of warfarin, calculated relative to starting position (t = 0). w1, w2, w3 refer to soluble simulations 1, 2 and 3 respectively. m1, m2, m3, refer to membrane simulations 1, 2 and 3, respectively. (TIF) [file pcbi.1003714.s011.tif]

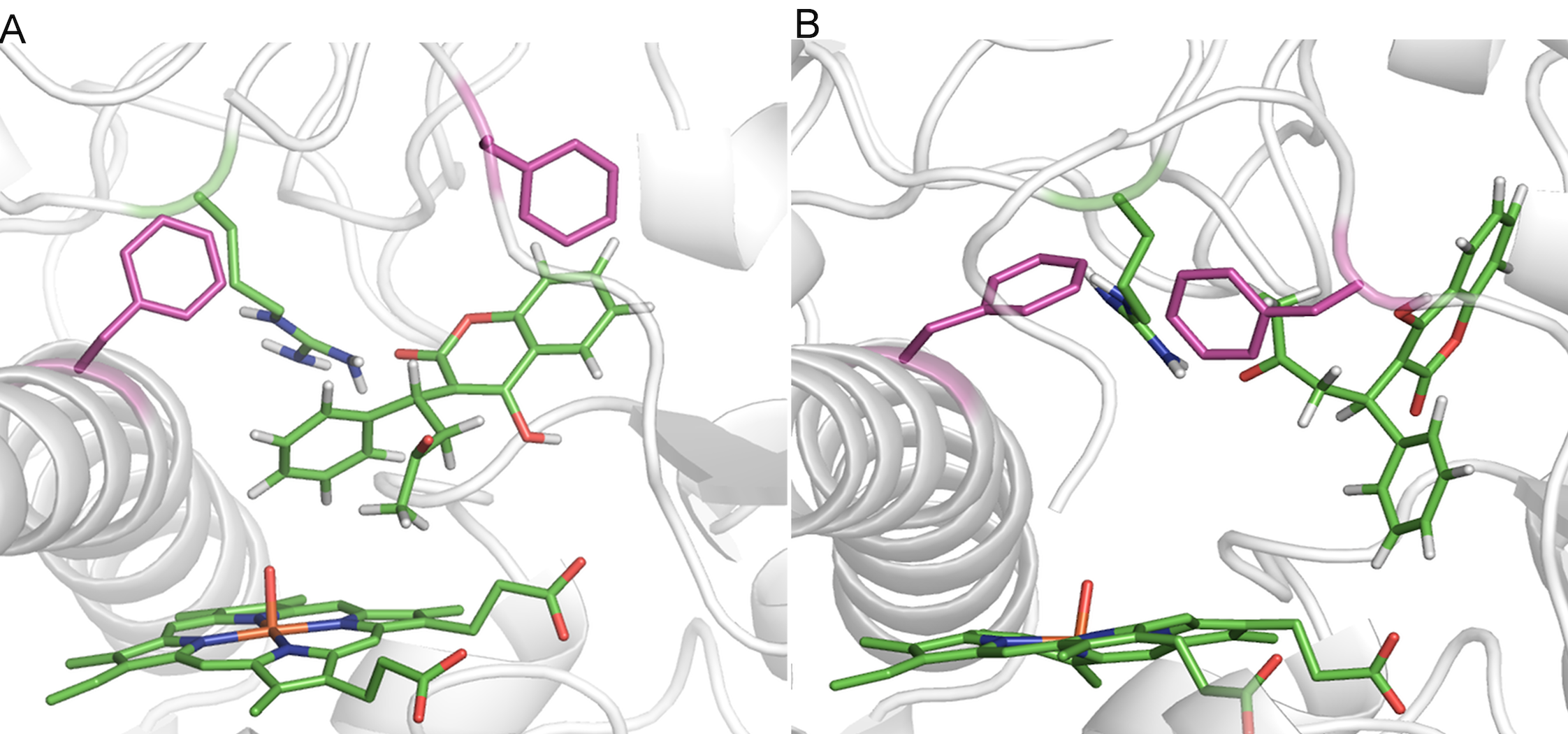

Supplement: Figure S12 — Structural snapshots from atomistic simulations of R-warfarin in the membrane-bound model of CYP3A4. Arg212 is shown in green and the gating residues Phe108 and Phe104 are displayed in purple. A. Structure from simulation 1 (after 30 ns). Arg212 forms a hydrogen bond to O2 of warfarin. The Phe108-Phe304 gate is open during this simulation. B. Structure from simulation 2 (after 45 ns). Arg212 forms a hydrogen bond to O4 of warfarin. The Phe108-Phe304 gate is closed during this simulation. (TIF) [file pcbi.1003714.s012.tif]

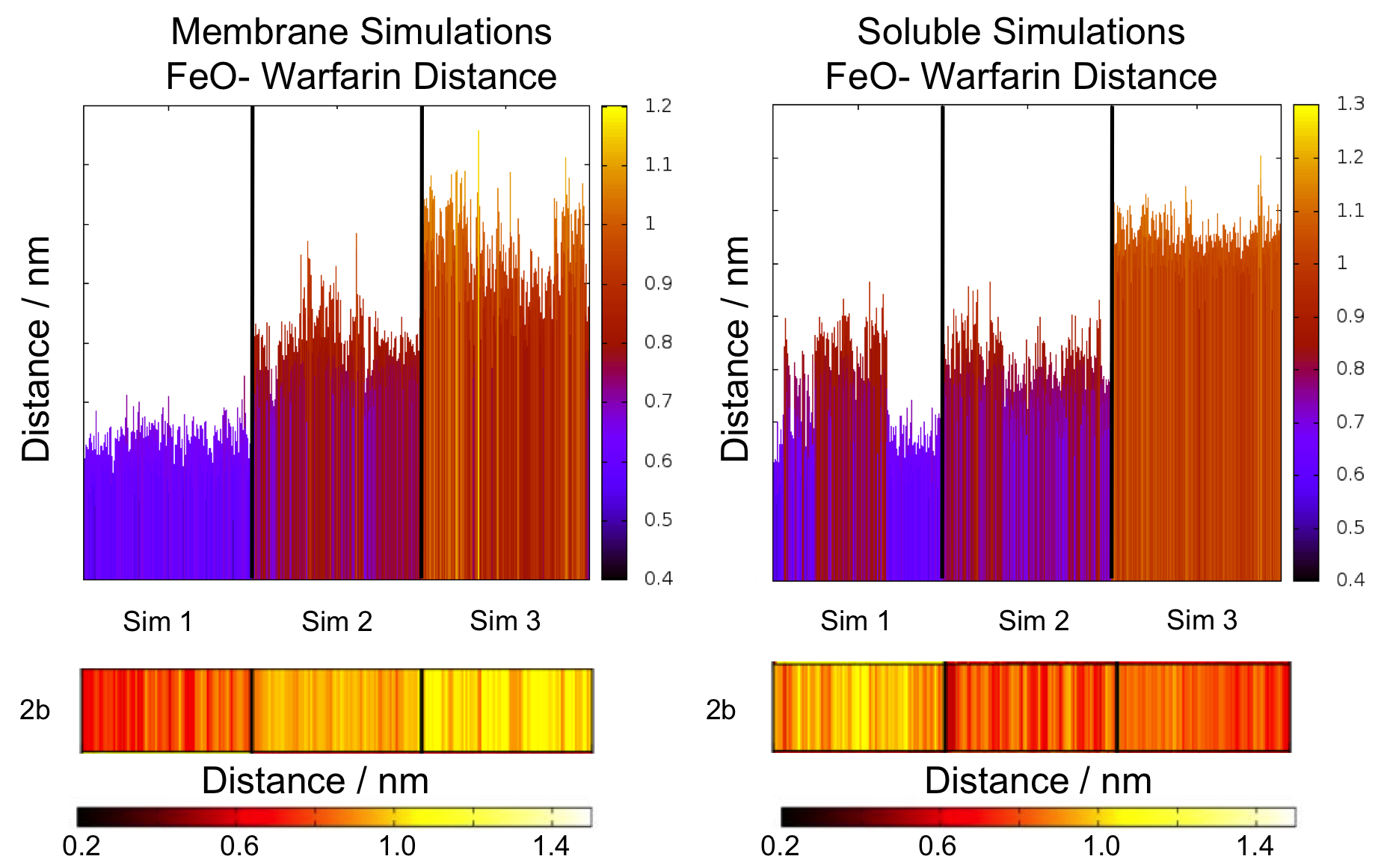

Supplement: Figure S13 — Correlation of warfarin motion gate 2b opening. The motion of warfarin (calculated in terms of the variation of the distance between the iron oxygen and the centre of mass of warfarin) within the active site increases with 2b gate opening. No correlation with other gates was found. (TIF) [file pcbi.1003714.s013.tif]

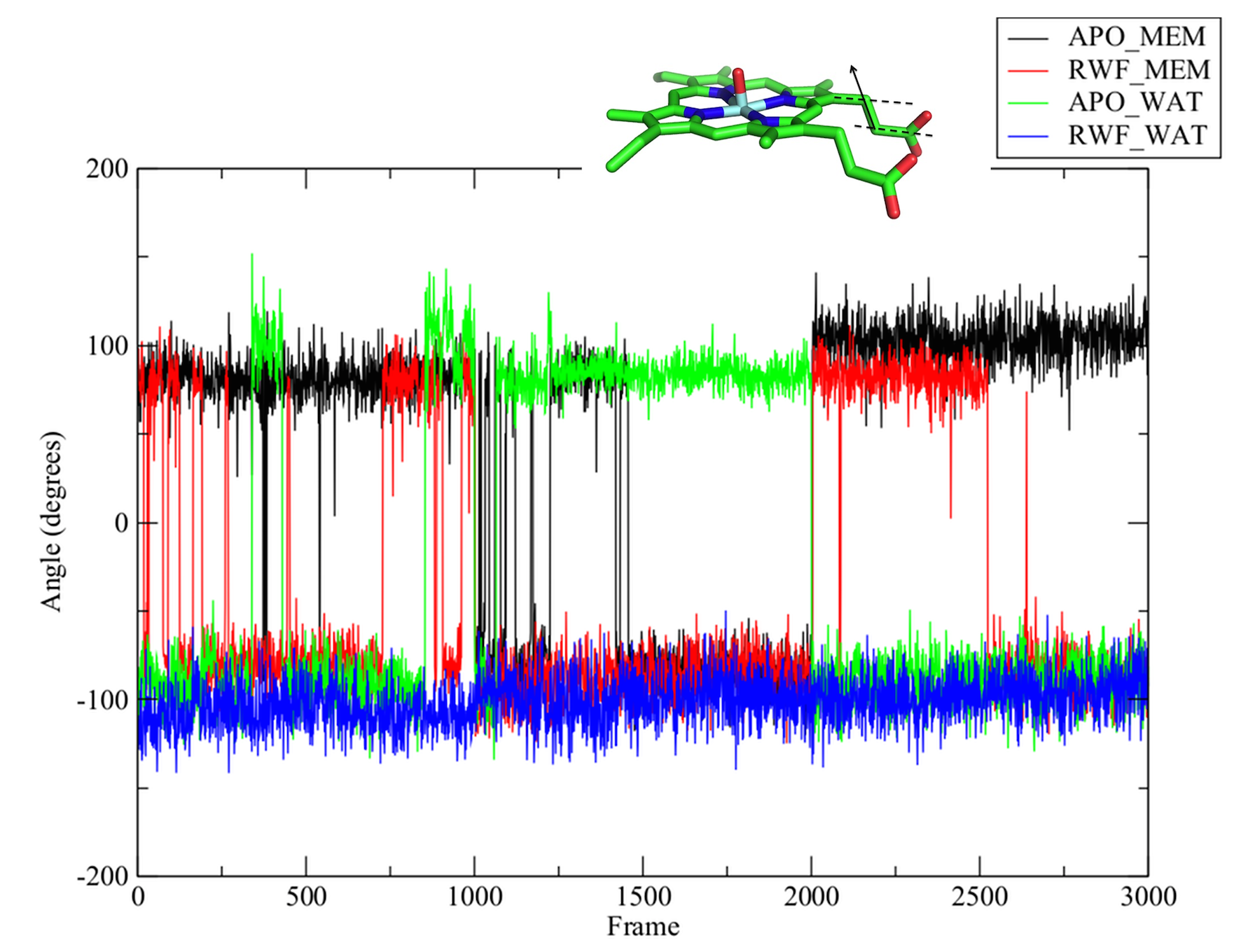

Supplement: Figure S14 — Heme propionate A dihedral angle (C3A-C2A-CAA-CBA) for all atomistic MD simulations. The dihedral angle value of +100° corresponds to the propionate being located on the distal side of the heme, a value of −100° corresponds to the propionate being in the proximal position. (TIF) [file pcbi.1003714.s014.tif]

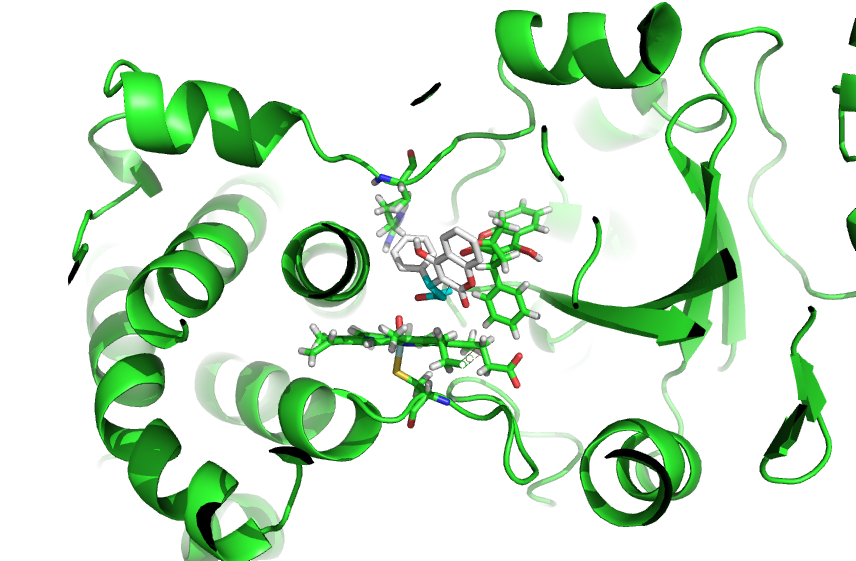

Supplement: Figure S15 — Docking result for multiple substrate binding with R-warfarin in CYP3A4. AUTODOCK VINA docking calculations were repeated using the R-warfarin-docked protein as the receptor input, and a second warfarin molecule as the ligand. The lowest-energy docked pose from this calculation places the second R-warfarin in the active site of CYP3A4. The second R-warfarin is shown in light grey. The atomistic model of the initial membrane-bound protein with a single R-warfarin molecule docked is shown in green. The heme group, Arg212, and Cys442 are shown in stick representation as in Figure 7. (TIF) [file pcbi.1003714.s015.tif]

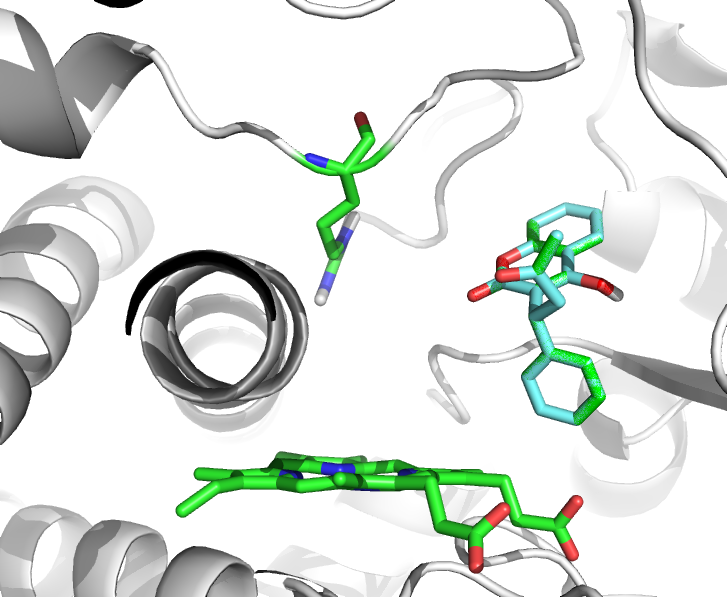

Supplement: Figure S16 — Docking pose selected as starting point for MD simulations of CYP3A4 with R-warfarin. Calculated using AUTODOCK VINA with the 1TQN crystal structure. The Arg212 residue (shown) was treated as flexible, the rest of the protein was treated as fixed. Nine docking poses were found, pose 8 (shown) was found to have the smallest distance between C10 (the position to undergo hydroxylation) and the heme iron. The calculated binding affinity of this pose is −8.5 kcal/mol. (TIFF) [file pcbi.1003714.s016.tiff]
