# Supplementary material for: A Multiscale Approach to Modelling Drug Metabolism by Membrane-Bound Cytochrome P450 Enzymes
Source: PLoS Comput Biol. 2014 Jul 17;10(7):e1003714. doi: 10.1371/journal.pcbi.1003714 (PMC4102395; doi:10.1371/journal.pcbi.1003714)
Supplement: Table S1 — Mean (and standard deviation) root mean squared deviations [in Å] of positions of protein backbone alpha carbon atoms from initial structure during entire MD simulations. Box dimensions for the production runs are shown in Å, as well as the total number of water molecules present in each simulation set-up. (DOCX) [file pcbi.1003714.s017.docx]

| **Simulation** | | **Mean RMSD (Standard deviation)** | **Box Dimensions (x, y, z)** | **Number of water molecules** | **Number of lipids** |
| --- | --- | --- | --- | --- | --- |
| **APO MEM** | **1** | 1.76 (0.49) | 117.41, 117.96, 160.40 | 54,214 | 330 POPC, 110 POPE |
|  | **2** | 1.77 (0.31) |  |  |  |
|  | **3** | 1.43 (0.23) |  |  |  |
| **RWF MEM** | **1** | 1.65 (0.25) | 119.62, 120.19, 154.52 | 54,214 | 330 POPC, 110 POPE |
|  | **2** | 1.40 (0.20) |  |  |  |
|  | **3** | 1.56 (0.31) |  |  |  |
| **APO WAT** | **1** | 2.35 (0.41) | 97.58, 97.58, 97.58 | 28,896 | n/a |
|  | **2** | 2.18 (0.24) |  |  |  |
|  | **3** | 1.86 (0.21) |  |  |  |
| **RWF WAT** | **1** | 1.98 (0.28) | 97.36, 97.36, 97.36 | 28,887 | n/a |
|  | **2** | 1.97 (0.21) |  |  |  |
|  | **3** | 1.89 (0.14) |  |  |  |

**Table S1. Mean (and standard deviation) root mean squared deviations [in Å] of positions of protein backbone alpha carbon atoms from initial structure during entire MD simulations. Box dimensions for the production runs are shown in Å, as well as the total number of water molecules present in each simulation set-up.**
