# Supplementary material for: A Multiscale Approach to Modelling Drug Metabolism by Membrane-Bound Cytochrome P450 Enzymes
Source: PLoS Comput Biol. 2014 Jul 17;10(7):e1003714. doi: 10.1371/journal.pcbi.1003714 (PMC4102395; doi:10.1371/journal.pcbi.1003714)
Supplement: Table S3 — Average values of the Mulliken atomic charges computed for the membrane-bound and solubilized CYP3A4 with R-warfarin bound (MR and SR, respectively) and without warfarin bound (MA and SA, respectively) optimized at the B3LYP-D:6-31G/CHARMM27 level of theory (average values are displayed in Figure 6A ). Calculated for 15 structures optimized at 5ns intervals over the last 20 ns of three 50 ns atomistic MD simulations. Standard deviations are given in parentheses. (DOCX) [file pcbi.1003714.s019.docx]

|  | Fe | O | Por | SMe |
| --- | --- | --- | --- | --- |
| M_R_ | 0.55 (0.03) | -0.41 (0.05) | -0.05 (0.06) | -0.09 (0.03) |
| M_A_ | 0.54 (0.04) | -0.45 (0.06) | -0.02 (0.07) | -0.07 (0.07) |
| S_R_ | 0.53 (0.03) | -0.41 (0.01) | -0.04 (0.05) | -0.08 (0.04) |
| S_A_ | 0.55 (0.03) | -0.45 (0.04) | 0.00 (0.06) | -0.10 (0.06) |

Table S3. Average values of the Mulliken atomic charges computed for the membrane-bound and solubilized CYP3A4 with R-warfarin bound (M_R_ and S_R_, respectively) and without warfarin bound (M_A_ and S_A_, respectively) optimized at the B3LYP-D:6-31G/CHARMM27 level of theory (average values are displayed in Figure 6A). Calculated for 15 structures optimized at 5ns intervals over the last 20 ns of three 50 ns atomistic MD simulations. Standard deviations are given in parentheses.
