# Supplementary material for: A Multiscale Approach to Modelling Drug Metabolism by Membrane-Bound Cytochrome P450 Enzymes
Source: PLoS Comput Biol. 2014 Jul 17;10(7):e1003714. doi: 10.1371/journal.pcbi.1003714 (PMC4102395; doi:10.1371/journal.pcbi.1003714)
Supplement: Table S4 — Percentage of gates that are open during atomistic MD simulations of membrane-bound (MR and MA) and solubilized (SR and SA) CYP3A4. The subscript labels R and A corresponds to the simulation models including and excluding R-warfarin, respectively. A gate is defined as open if the distance between the centre of mass of the two gating residue side chains is less than 7 Å. (DOCX) [file pcbi.1003714.s020.docx]

|  | Percentage of gates open |
| --- | --- |
| M_R_ | 40.9 ± 2.3 |
| M_A_ | 40.8 ± 2.2 |
| S_R_ | 31.6 ± 2.6 |
| S_A_ | 61.0 ± 2.1 |

**Table S4 Percentage of gates that are open during atomistic MD simulations of membrane-bound (M_R_ and M_A_) and solubilized (S_R_ and S_A_) CYP3A4.** The subscript labels R and A corresponds to the simulation models including and excluding R-warfarin, respectively. A gate is defined as open if the distance between the centre of mass of the two gating residue side chains is less than 7 Å.
